# Supplementary material for: Dual LSD1 and HDAC6 Inhibition Induces Doxorubicin Sensitivity in Acute Myeloid Leukemia Cells
Source: Cancers (Basel). 2022 Dec 6;14(23):6014. doi: 10.3390/cancers14236014 (PMC9737972; doi:10.3390/cancers14236014)
Supplement: Supplementary file 1 [file cancers-14-06014-s001.zip › cancers-1813561-supplementary.pdf]

cancers

MDPI

Supplementary Material: Dual LSD1 and HDAC6 inhibition induces doxorubicin sensitivity in acute myeloid leukemia cells

Ipek Bulut, Adam Lee, Buse Cevatemre, Dusan Ruzic, Roman Belle, Akane Kawamura, Sheraz Gul, Katarina Nikolic, A. Ganesan and Ceyda Acilan

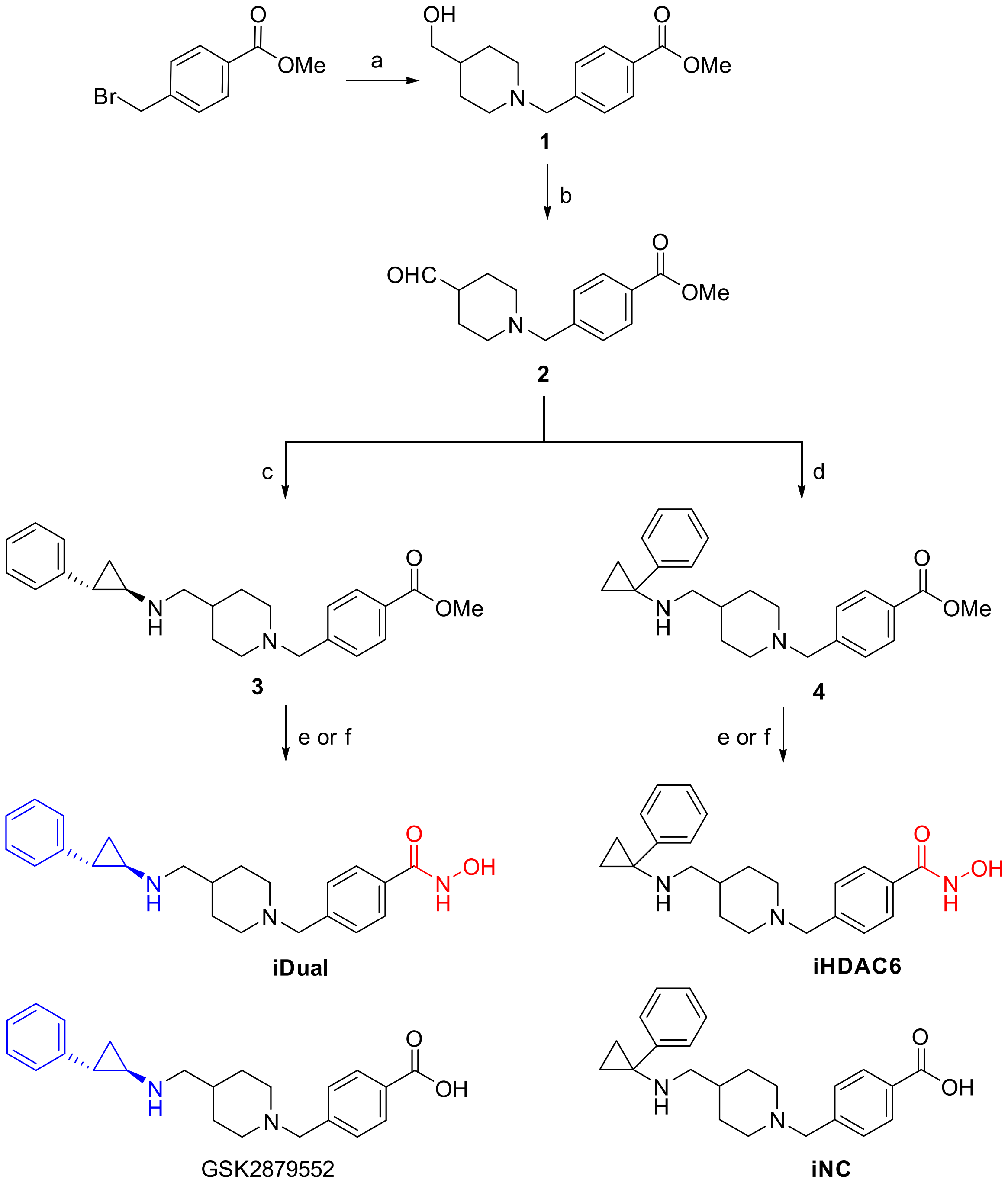

Figure S1. Scheme for the synthesis of iDual and the controls iHDAC6 and iNC.

Text S1: Compound synthesis

Methyl 4-{[4-(hydroxymethyl)piperidin-1-yl]methyl}benzoate (**1**). A flame dried flask was charged with methyl *p*-bromomethylbenzoic acid (3.00 g, 13.11 mmol) and 4-piperidine-methanol (1.51 g, 13.09 mmol). Anhydrous acetonitrile (60 mL) was then added, making a white suspension. Potassium carbonate (5.43 g, 39.29 mmol) was added, and the mixture refluxed under a nitrogen atmosphere for 2 h. The mixture was allowed to cool to room temperature before being filtered and concentrated under reduced pressure. The concentrate was dissolved in EtOAc (50 mL) and 1 M HCl (50 mL) added. The aqueous layer was separated and washed with EtOAc (50 mL) and then basified with 4 M NaOH to pH 10. The aqueous mixture was then twice extracted with EtOAc (50 mL) and the combined organic layers washed with brine (50 mL) and dried over $MgSO_4$. Solvent removal in vacuo gave **1** (2.86 g, 83%) as a white solid. Mp 57-58 ºC; ^{1}H NMR (400 MHz, $CDCl_3$): δ ppm 7.98 (d, *J* = 8.3 Hz, 2H), 7.39 (d, *J* = 8.4 Hz, 2H), 3.90 (s, 3H), 3.55 (s, 2H), 3.48 (d, *J* = 6.4 Hz, 2H), 2.88 (d, *J* = 11.5 Hz, 2H), 1.99 (td, *J* = 11.7, 2.4 Hz, 2H), 1.74–1.68 (m, 2H), 1.55–1.45 (m, 2H), 1.31 (qd, *J* = 12.3, 12.0, 12.0, 3.9 Hz, 2H); ^{13}C NMR (101 MHz, $CDCl_3$): δ ppm 167.1, 143.8, 129.6, 129.1, 129.0, 67.8, 63.0, 53.6, 52.1, 38.5, 28.7; ESI-MS *m/z* calcd for $C_{15}H_{21}NO_3$ $[M+H]^+$ 264.1594, found 264.1596.

Methyl 4-[(4-formylpiperidin-1-yl)methyl]benzoate (**2**). DMSO (4.4 mL, 61.9 mmol) was dissolved in dry dichloromethane (100 mL) and the solution cooled to −78 °C. Oxalyl chloride (3.4 mL, 39.6 mmol) was then also dissolved in dry dichloromethane (38 mL) and cooled to −78 °C before being slowly added to the DMSO solution. The mixture was then stirred for 30 minutes at -78 ºC under a nitrogen atmosphere before **2** (7.01 g, 26.6 mmol) dissolved in dry dichloromethane (40 mL) and cooled to −78 °C was slowly added. The resulting mixture was stirred at −78 °C under nitrogen for 3 hours. After this time, trimethylamine (19 mL, 136.3 mmol) dissolved in dry dichloromethane (20 mL) and cooled to −78 °C was added and the reaction mixture stirred at −78 °C for a further 20 minutes. Water (50 mL) was added and the mixture allowed to warm to room temperature. The aqueous layer was separated, and the pH adjusted to ~7 with 2 M HCl. The aqueous layer was then extracted with dichloromethane (20 mL) and all organic layers combined and washed with water and brine (50 mL), dried over $MgSO_4$ and concentrated. The concentrate was then purified on a silica column eluting with a gradient of 0–100% EtOAc in petroleum ether to give **2** (4.9 g, 71%) as a yellow oil which solidified to a crystalline solid under high vacuum. Mp 49–50 °C; ^{1}H NMR (400 MHz, $CDCl_3$): δ ppm 9.64 (d, *J* = 1.2 Hz, 1H), 7.97 (d, *J* = 8.4 Hz, 2H), 7.37 (d, *J* = 8.5 Hz, 2H), 3.89 (s, 3H), 3.53 (s, 2H), 2.80–2.75 (m, 2H), 2.28–2.20 (m, 1H), 2.11 (td, *J* = 11.1, 2.7 Hz, 2H), 1.90–1.85 (m, 2H), 1.73–1.63 (m, 2H); ^{13}C NMR (101 MHz, $CDCl_3$): δ ppm 203.9, 167.1, 144.0, 129.6, 129.0, 128.8, 62.9, 52.7, 52.1, 47.9, 25.5; ESI MS *m/z* calcd for $C_{15}H_{19}NO_3$ $[M+H]^+$ 262.1438, found 262.1441.

Methyl4-{[4-({[(trans)-2-phenylcyclopropyl]amino}methyl)piperidin-1-yl]methyl} benzoate (**3**). To a solution of **2** (4.10 g, 15.70 mmol) in dry methanol (33 mL) was added *trans*-2-phenylcyclopropanamine (tranylcypromine, 2.50 g, 18.78 mmol). The mixture was heated to reflux for 10 min before cooling back to room temperature. Sodium borohydride (0.89 g, 23.53 mmol) was then slowly added and the mixture stirred at room temperature for 1 hour. Water (50 mL) was added followed by dichloromethane (50 mL) and the organic layer separated and washed with a solution of 10% acetic acid (50%). Brine (50 mL) was then slowly added allowing the precipitation of a white solid. The solid was filtered and then suspended in propan-2-ol and sonicated before being filtered again to give **3** (2.06 g, 35%) as a white solid. ^{1}H NMR (400 MHz, Methanol-d_4): δ ppm 8.11 (d, *J* = 8.3 Hz, 2H), 7.71 (d, *J* = 8.4 Hz, 2H), 7.33–7.27 (m, 2H), 7.25–7.16 (m, 3H), 4.37 (s, 2H), 3.93 (s, 3H), 3.46 (d, *J* = 12.8 Hz, 2H), 3.17 (d, *J* = 6.9 Hz, 2H), 3.07 (t, *J* = 12.5 Hz, 2H), 2.98 (ddd, *J* = 7.9, 4.4, 3.6 Hz, 1H), 2.58 (ddd, *J* = 10.3, 6.6, 3.6 Hz, 1H), 2.23–2.02 (m, 3H), 1.70 (q, *J* = 12.9 Hz, 2H), 1.59 (ddd, *J* = 10.4, 6.7, 4.4 Hz, 1H), 1.36 (dt, *J* = 7.8, 6.6 Hz, 1H); ^{13}C NMR (101 MHz, DMSO-d_6): δ ppm 165.8, 138.9, 134.9, 131.9, 130.4, 129.3, 128.4, 126.4, 126.4, 58.1, 52.3, 51.6, 50.6, 37.9, 30.5, 26.5, 20.3, 12.4; ESI-MS *m/z* calcd for $C_{24}H_{30}N_2O_2$ $[M+H]^+$ 379.2380, found 379.2380.

Methyl 4-{[4-({[1-phenylcyclopropyl]amino}methyl) piperidin-1-yl]methyl} benzoate (**4**). 1-Phenyl-1-cyclopropylamine (0.50 g, 2.95 mmol) and **2** (0.68 g, 2.46 mmol) were dissolved in anhydrous methanol (5 mL) and the mixture placed under reflux for 1 hour before being cooled to room temperature. $NaBH_4$ (0.13 g, 3.69 mmol) was slowly added, and the reaction mixture stirred at ambient temperature for 1 hour, followed by quenching with water (50 mL). The mixture was extracted with dichloromethane (3 × 50mL) and the combined organic extracts dried over $MgSO_4$ and filtered. The filtrate was concentrated and purified on a silica column eluting with a gradient of 0-10% MeOH in dichloromethane to give **4** (0.81 g, 87%) as a yellow oil. ^{1}H NMR (400 MHz, $CDCl_3$): δ ppm 7.89 (d, *J* = 8.3 Hz, 2H), 7.30 (d, *J* = 8.0 Hz, 2H), 7.26–7.18 (m, 4H), 7.15–7.10 (m, 1H), 3.83 (s, 3H), 3.43 (s, 2H), 2.74 (d, *J* = 11.4 Hz, 2H), 2.34 (d, *J* = 6.5 Hz, 2H), 1.85 (td, *J* = 11.5, 2.5 Hz, 2H), 1.57 (dd, *J* = 13.1, 2.9 Hz, 2H), 1.29–1.16 (m, 1H), 1.14–1.11 (m, 2H), 0.90–0.85 (m, 1H), 0.85– 0.80 (m, 1H); ^{13}C NMR (101 MHz, $CDCl_3$): δ ppm 167.2, 143.8, 129.6, 129.0, 128.9, 128.3, 127.4, 126.3, 63.1, 53.9, 52.4, 52.1, 42.5, 36.6, 30.7, 15.9; ESI-MS *m*/*z* calcd for $C_{24}H_{30}N_2O_2$ $[M+H]^+$ 379.24, found 379.24.

GSK2879552. Methyl ester **3** (0.50 g, 1.32 mmol) was dissolved in a 3:1 mixture of THF:H_2O and to this was added LiOH (0.10 g, 4.18 mmol). The reaction mixture was stirred at 50 ºC for 16 hours and then allowed to cool to room temperature before adjusting to pH 2 with saturated $KHSO_4$. It was then concentrated under reduced pressure and the residue purified on an amine functionalized silica column eluting with a gradient of 10-100% EtOAc in petroleum ether to give GSK2879552 (0.20 g, 41%) as a white solid. ^{1}H NMR (400 MHz, Methanol-*d*4, HCl salt): δ ppm 8.12 (d, *J* = 8.3 Hz, 2H), 7.70 (d, *J* = 8.4 Hz, 2H), 7.33–7.28 (m, 2H), 7.26–7.16 (m, 3H), 4.43 (s, 2H), 3.54 (d, *J* = 12.3 Hz, 2H), 3.26–3.07 (m, 4H), 3.02 (dt, *J* = 7.9, 4.1 Hz, 1H), 2.61 (ddd, *J* = 10.3, 6.6, 3.6 Hz, 1H), 2.27–2.06 (m, 3H), 1.81–1.57 (m, 3H), 1.38 (dt, *J* = 7.9, 6.7 Hz, 1H); ^{13}C NMR (101 MHz, Methanol-*d*4): δ ppm 167.3, 137.9, 133.5, 132.3, 131.2, 130.0, 128.3, 126.6, 126.0, 59.6, 52.0, 51.5, 38.3, 30.8, 26.7, 21.0, 12.0; ESI-MS *m*/*z* calcd for $C_{23}H_{28}N_2O_2$ $[M+H]^+$ 365.2224, found 365.2228.

N-Hydroxy-4-[(4-{[(2-phenylcyclopropyl)amino]methyl}piperidin-1-yl)methyl]benzamide (**iDual**). In separate flasks, **3** (1.00 g, 2.64 mmol), hydroxylamine hydrochloride (0.97 g, 13.96 mmol) and potassium hydroxide (1.52 g, 27.09 mmol) were dissolved in dry methanol (25 mL, 5 mL and 8 mL respectively). The potassium hydroxide solution was then slowly added to the hydroxylamine solution and the resulting mixture stirred at room temperature for 30 minutes. This mixture was then filtered, and the filtrate added to the benzoate solution, which was then stirred at room temperature overnight. Reaction completion was confirmed via TLC and the reaction mixture then adjusted to pH 7 with 6 M HCl, followed by filtration and concentration. The concentrate was purified using a C18 reverse phase column, eluting with a gradient of 10–100% methanol in water to give **iDual** (0.25 g, 25%, HPLC purity 98%) as a white solid. Mp 57-58 °C; IR (cm^{-1}) 2915, 1611; ^{1}H NMR (400 MHz, DMSO-*d*6): δ ppm 7.69 (d, *J* = 8.3 Hz, 2H), 7.34 (d, *J* = 8.2 Hz, 2H), 7.21 (t, *J* = 7.5 Hz, 2H), 7.13–7.06 (m, 1H), 7.03–6.99 (m, 2H), 3.45 (s, 2H), 2.82–2.70 (m, 2H), 2.46 (dd, *J* = 6.6, 2.2 Hz, 2H), 2.17 (ddd, *J* = 7.3, 4.4, 3.0 Hz, 1H), 1.88 (tdd, *J* = 11.5, 4.5, 2.3 Hz, 2H), 1.74 (ddd, *J* = 9.0, 5.4, 2.7 Hz, 1H), 1.70–1.59 (m, 2H), 1.36 (ddt, *J* = 11.1, 7.5, 3.9 Hz, 1H), 1.11 (dddd, *J* = 15.5, 11.8, 7.6, 3.6 Hz, 2H), 0.97–0.87 (m, 2H); ^{13}C NMR (101 MHz, Methanol-*d*4): δ ppm 175.3, 143.4, 140.5, 138.2, 130.2, 130.1, 129.2, 126.7, 126.5, 64.0, 56.2, 54.5, 49.8, 42.6, 36.5, 31.1, 25.1, 16.6; ESI-MS *m*/*z* calcd for $C_{23}H_{29}N_3O_2$ $[M+H]^+$ 380.2333, found 380.2334.

*N-Hydroxy-4-[(4-{[(1-phenylcyclopropyl)amino]methyl}piperidin-1-yl)methyl]benzamide (**iHDAC6**).* In separate flasks, **4** (1.00 g, 2.64 mmol), hydroxylamine hydrochloride (0.85, 13.21 mmol) and potassium hydroxide (1.54 g, 26.42 mmol) were dissolved in dry methanol (15 mL, 10 mL and 10 mL respectively). The potassium hydroxide solution was then slowly added to the hydroxylamine solution and the resulting mixture stirred at room temperature for 30 min. This mixture was then filtered, and the filtrate added to the benzoate solution and stirred at room temperature overnight. Reaction completion was confirmed by TLC and the pH then adjusted to 7 with 6 M HCl. The reaction mixture was

filtered and concentrated. The concentrate was then dissolved in cold ethanol and again filtered and concentrated. The concentrate was purified using an amine functionalised column, eluting with a gradient of 0–10% methanol in dichloromethane to give **iHDAC6** (0.30 g, 30%, HPLC purity 98%) as a white solid. ^{1}H NMR (400 MHz, Methanol-d_4): δ ppm 7.69 (d, *J* = 8.3 Hz, 2H), 7.39 (d, *J* = 8.4 Hz, 2H), 7.36–7.24 (m, 4H), 7.21–7.14 (m, 1H), 3.53 (s, 2H), 2.83 (d, *J* = 11.8 Hz, 2H), 2.35 (d, *J* = 6.7 Hz, 2H), 1.98 (td, *J* = 11.8, 2.5 Hz, 2H), 1.71–1.63 (m, 2H), 1.41–1.30 (m, 1H), 1.20–1.06 (m, 2H), 1.00–0.91 (m, 2H), 0.91–0.84 (m, 2H); ^{13}C NMR (101 MHz, Methanol-d_4): δ ppm 196.1, 172.2, 170.7, 160.7, 159.0, 158.5, 157.4, 157.1, 157.1, 156.2, 155.7, 91.9, 82.6, 81.4, 71.7, 65.1, 59.3, 43.3; ESI-MS *m*/*z* calcd for $C_{23}H_{29}N_3O_2$ $[M+H]^+$ 380.2333, found 380.2330.

4-[(4-{[(1-Phenylcyclopropyl)amino]methyl}piperidin-1-yl)methyl]benzoic acid (**iNC**). Ester **4** (0.40 g, 1.06 mmol) was dissolved in a 3:1 mixture of THF:H_2O. LiOH (0.08 g, 3.17 mmol) was added and the solution stirred at 50 °C for 16 h. The mixture was then allowed to cool to room temperature before adjusting to pH 2 with saturated $KHSO_4$. It was then concentrated under reduced pressure and the concentrate purified on an amine functionalized silica column eluting with a gradient of 10–100% EtOAc in petroleum ether to give **iNC** (0.81 g, 87%, HPLC purity 98%) as a white solid. ^{1}H NMR (400 MHz, Methanol-d_4): δ ppm 7.90 (d, *J* = 8.3 Hz, 2H), 7.36–7.26 (m, 6H), 7.21–7.16 (m, 1H), 3.56 (s, 1H), 2.89 (d, *J* = 12.0 Hz, 2H), 2.36 (d, *J* = 6.7 Hz, 2H), 2.03 (td, *J* = 11.9, 2.6 Hz, 2H), 1.73–1.65 (m, 2H), 1.43–1.32 (m, 1H), 1.20–1.09 (m, 2H), 0.99–0.92 (m, 2H), 0.92–0.84 (m, 2H); ^{13}C NMR (101 MHz, Methanol-d_4): δ ppm 175.1, 144.1, 139.9, 138.3, 130.2, 129.2, 128.9, 127.5, 63.8, 54.3, 53.2, 43.5, 36.9, 31.0, 15.2; ESI-MS *m*/*z* calcd for $C_{23}H_{28}N_2O_2$ $[M+H]^+$ 365.2224, found 365.2218.

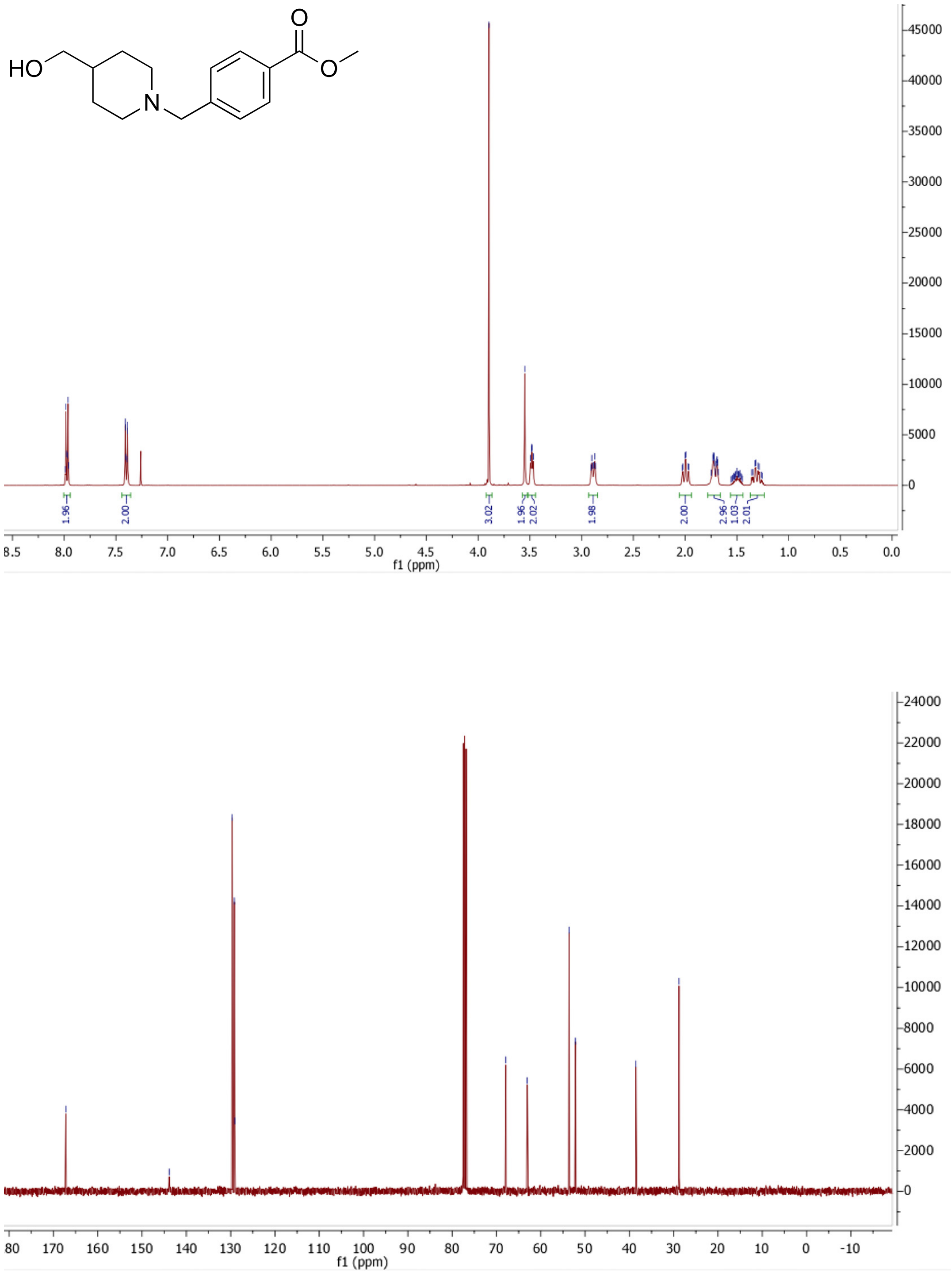

Figure S2. ^{1}H and ^{13}C NMR spectra of **1**.

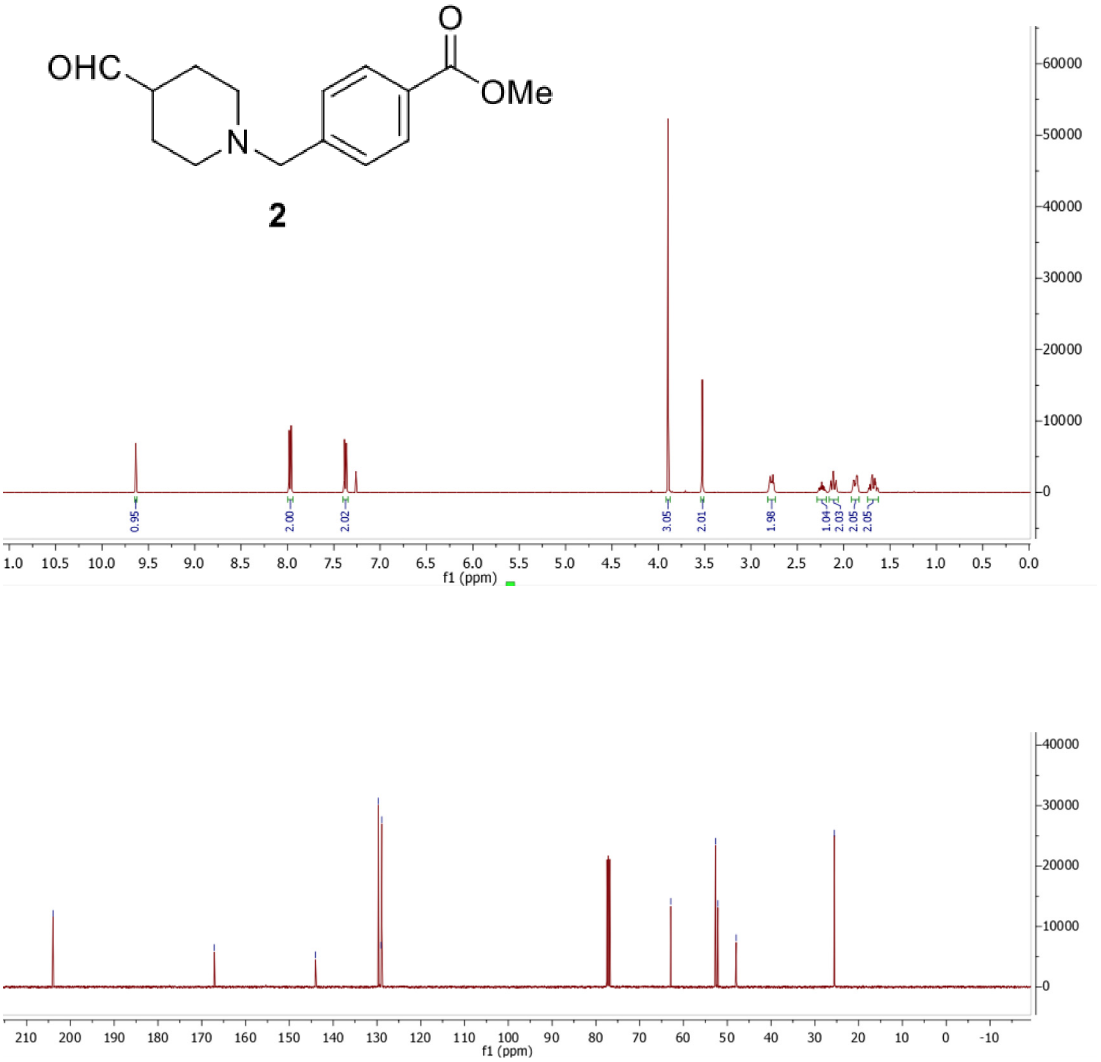

Figure S3. ^{1}H and ^{13}C NMR spectra of **2.**

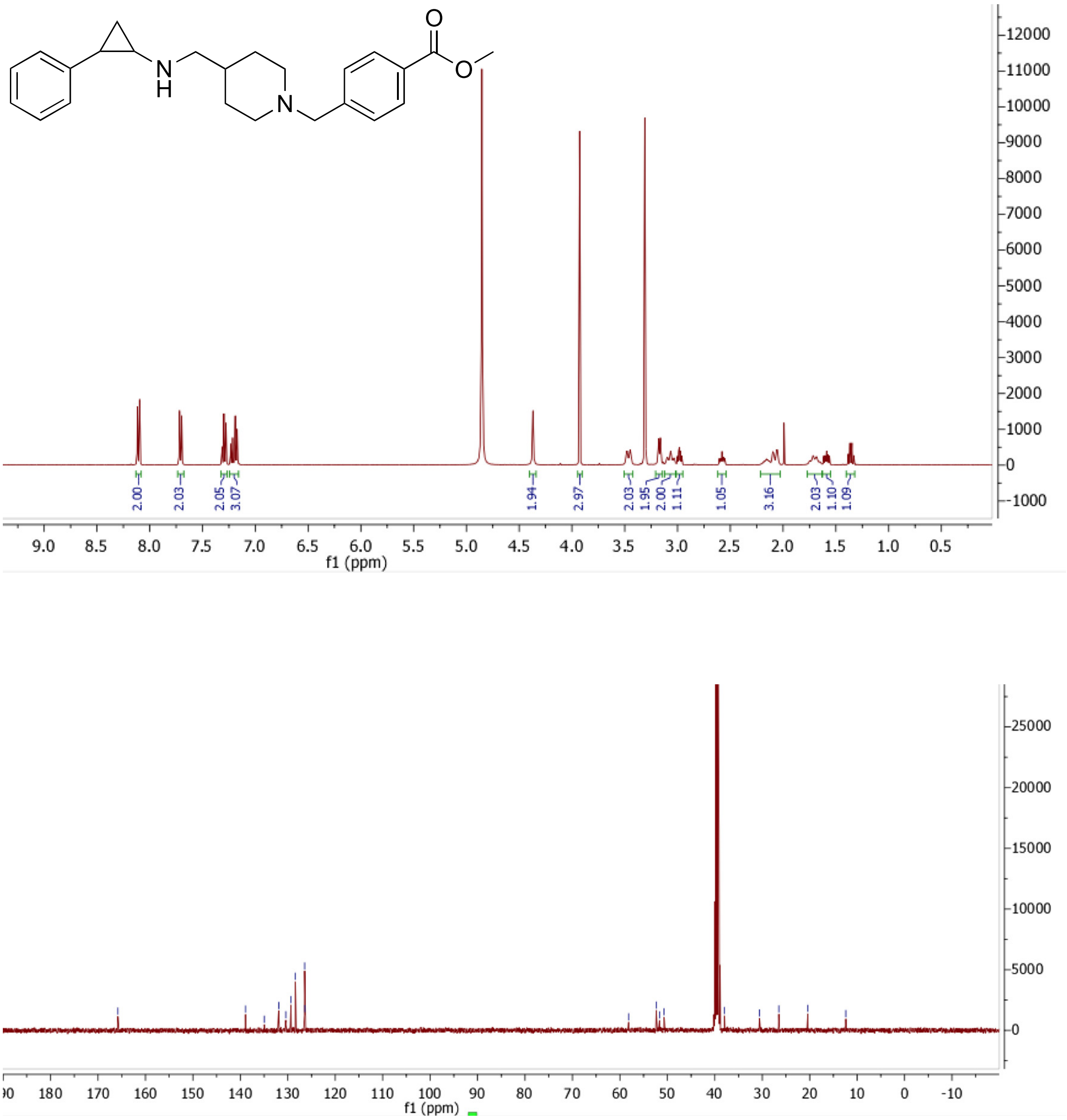

Figure S4. ^{1}H and ^{13}C NMR spectra of **3**.

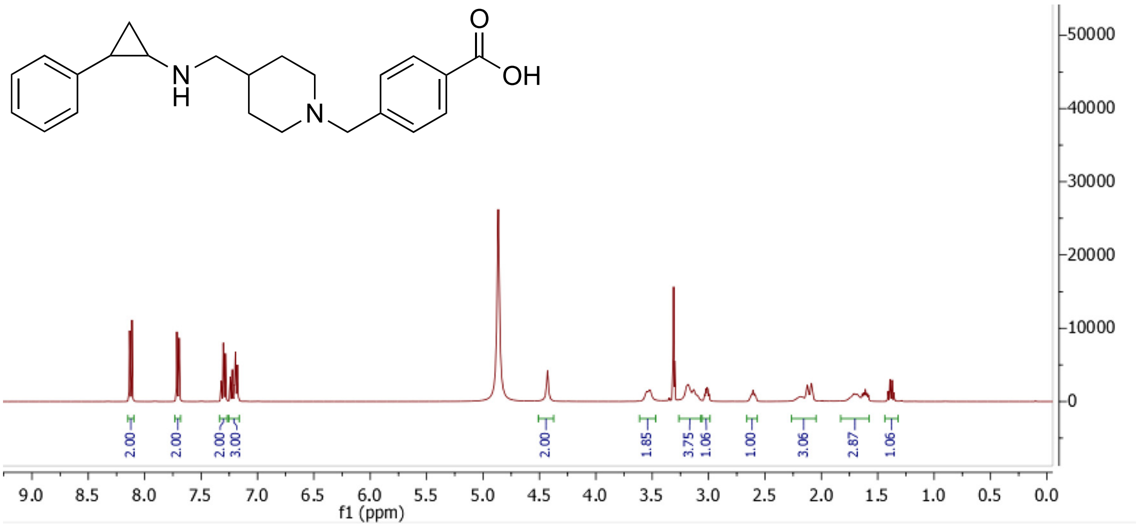

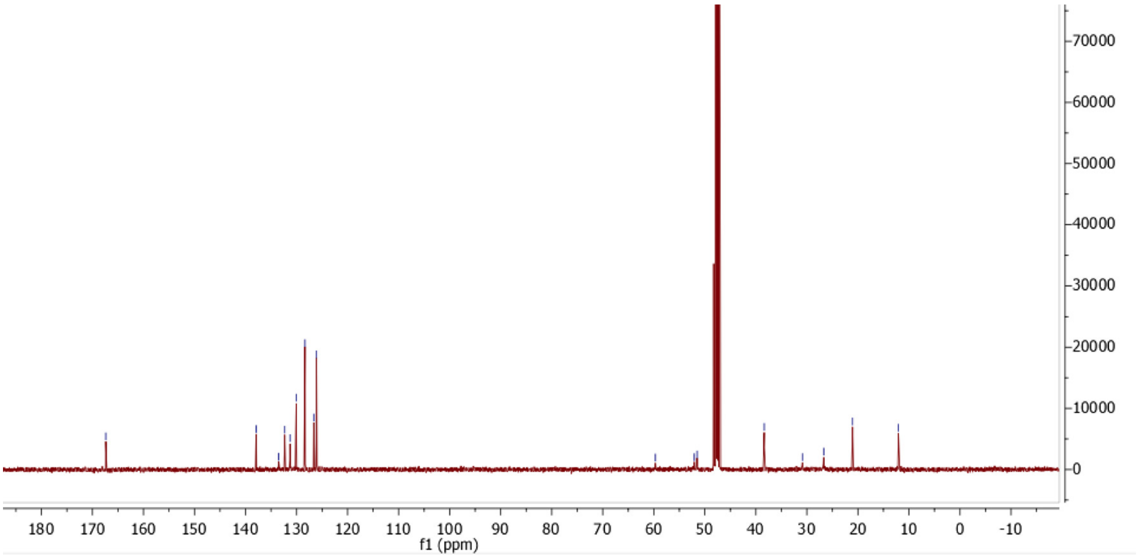

Figure S5. ^{1}H and ^{13}C NMR spectra of GSK2879552.

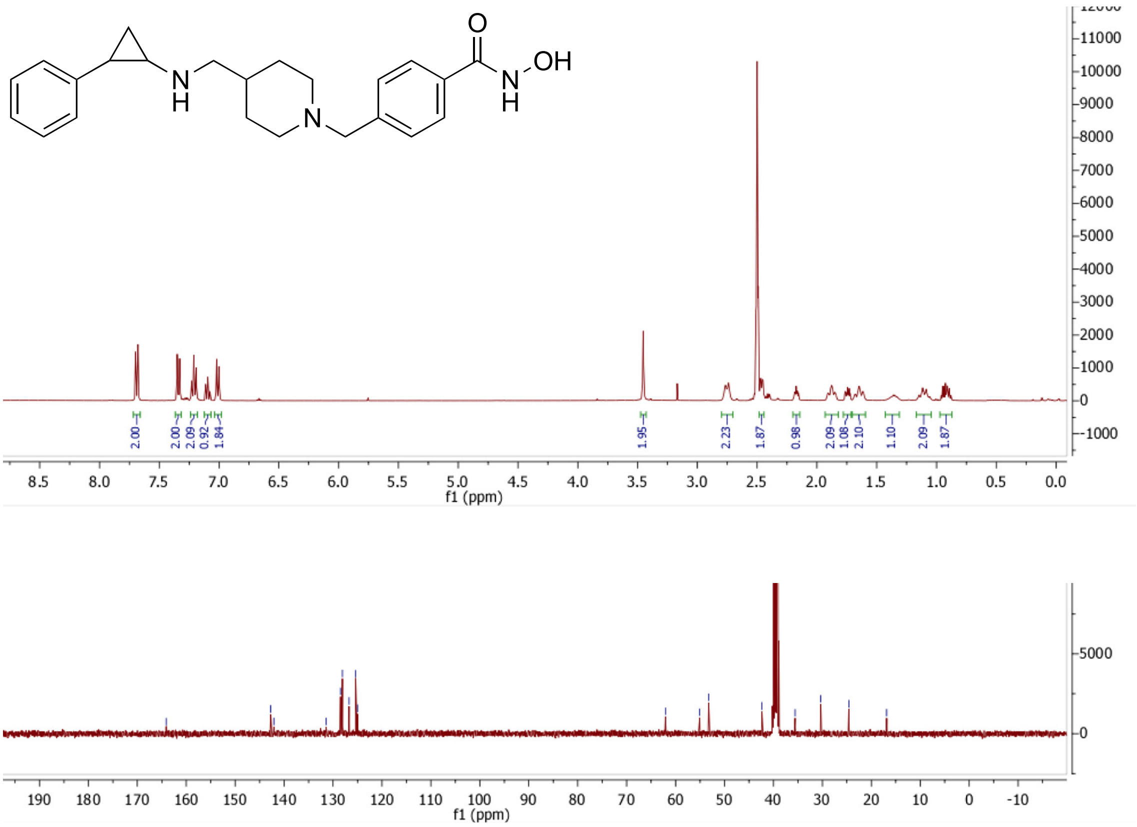

Figure S6. ^{1}H and ^{13}C NMR spectra of iDual.

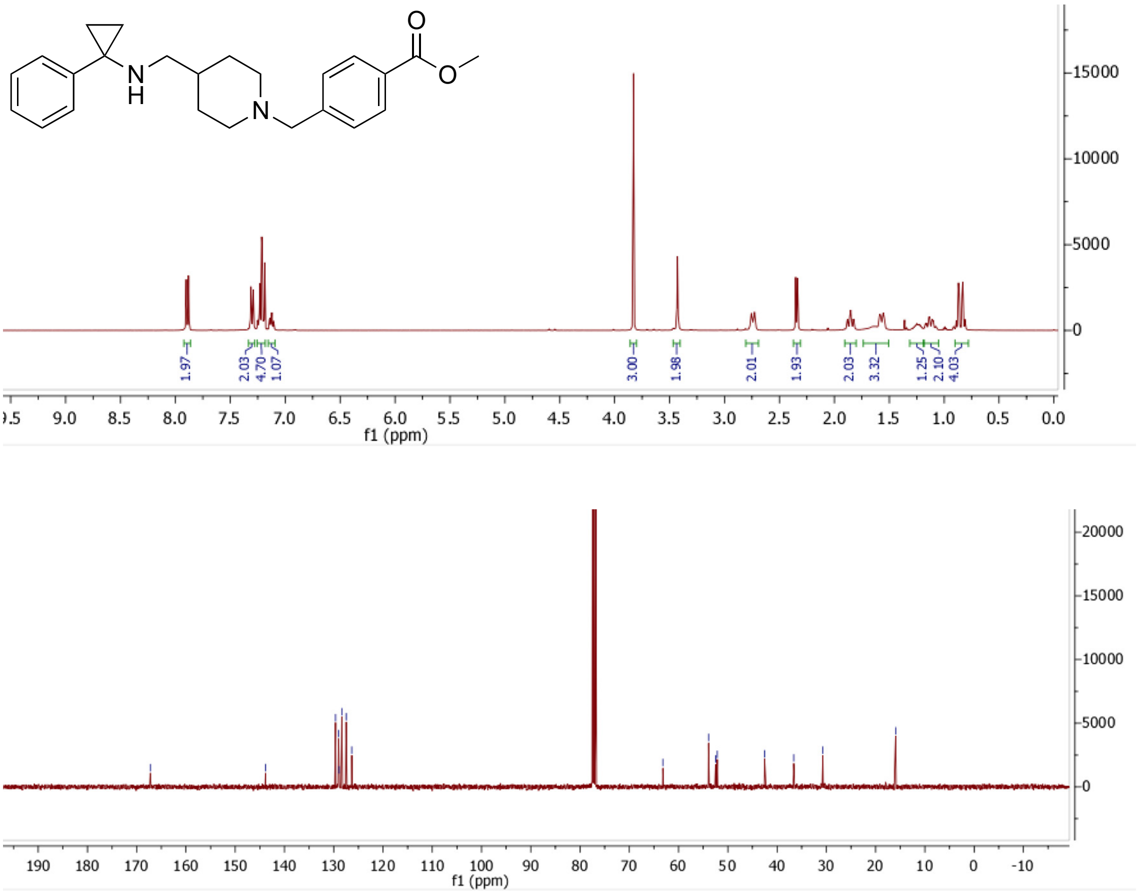

Figure S7. ^{1}H and ^{13}C NMR spectra of **4**.

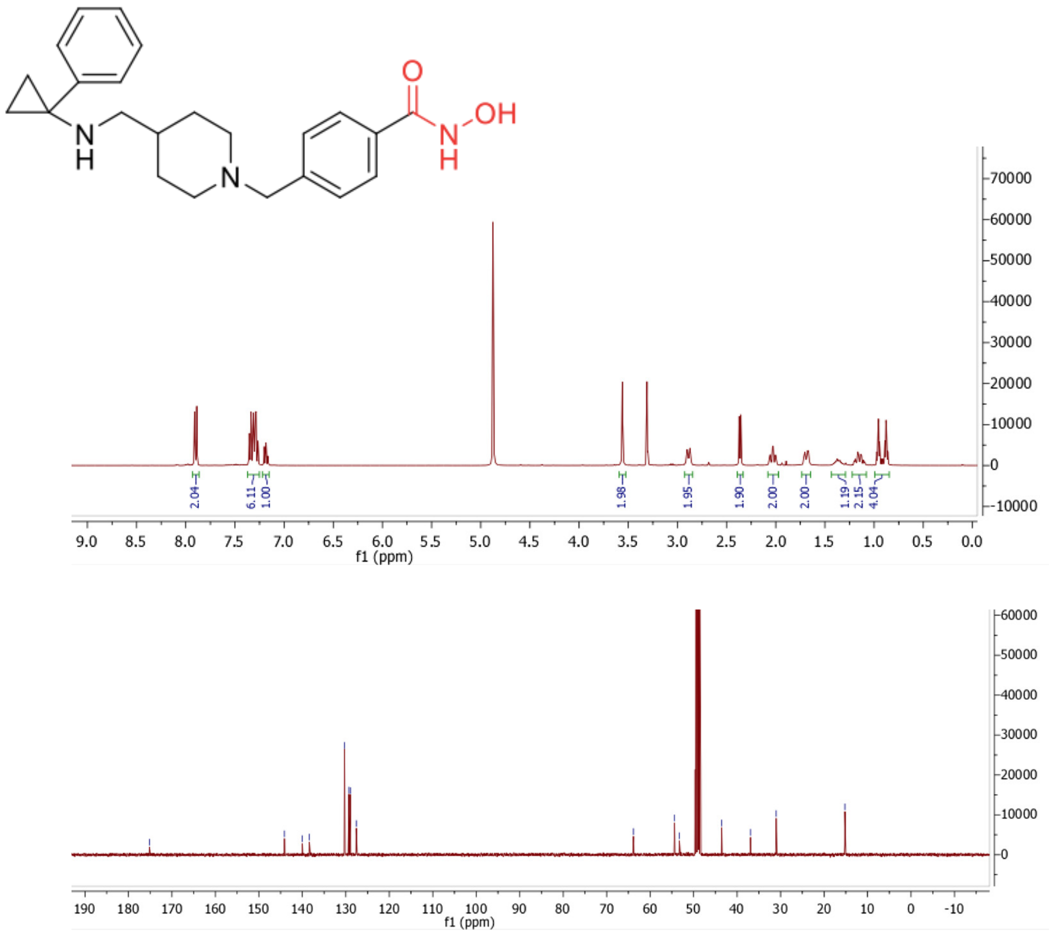

Figure S8. ^{1}H and ^{13}C NMR spectra of iHDAC6.

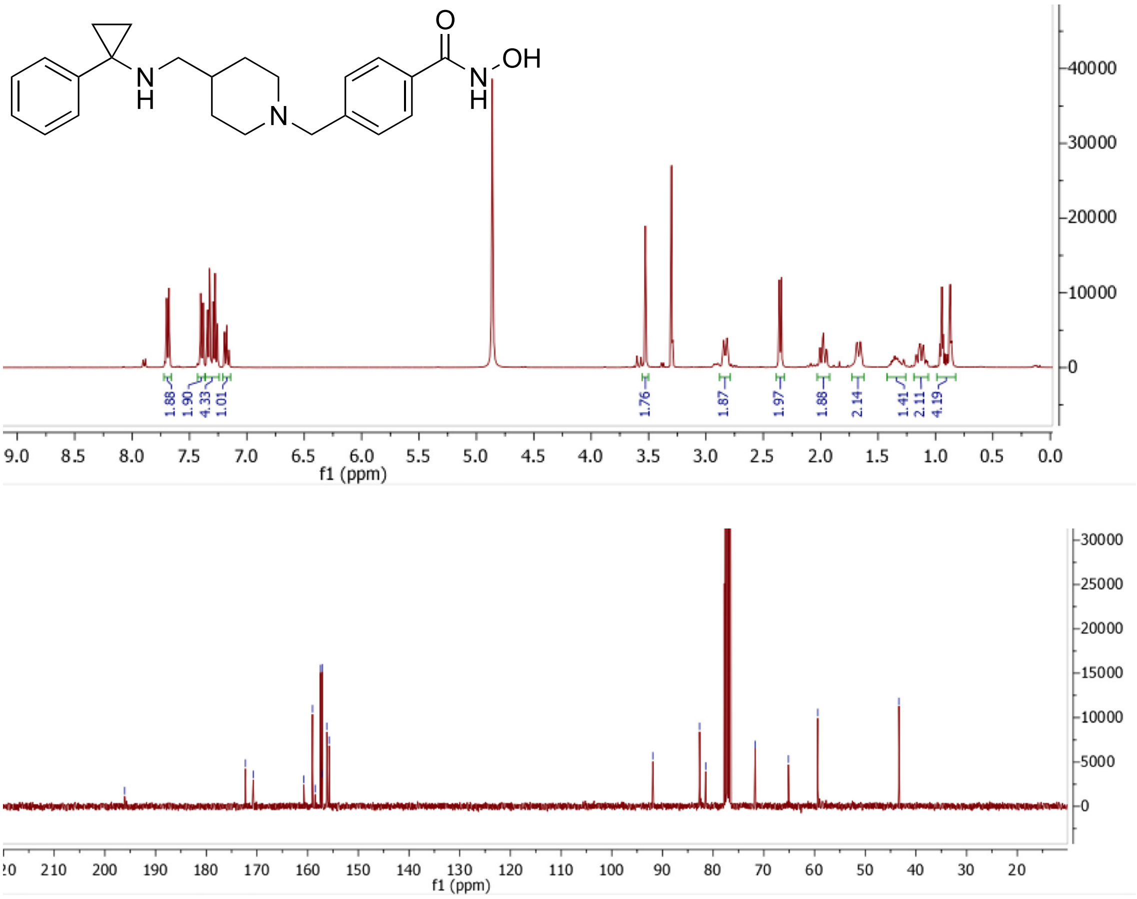

Figure S9. ^{1}H and ^{13}C NMR spectra of iNC.

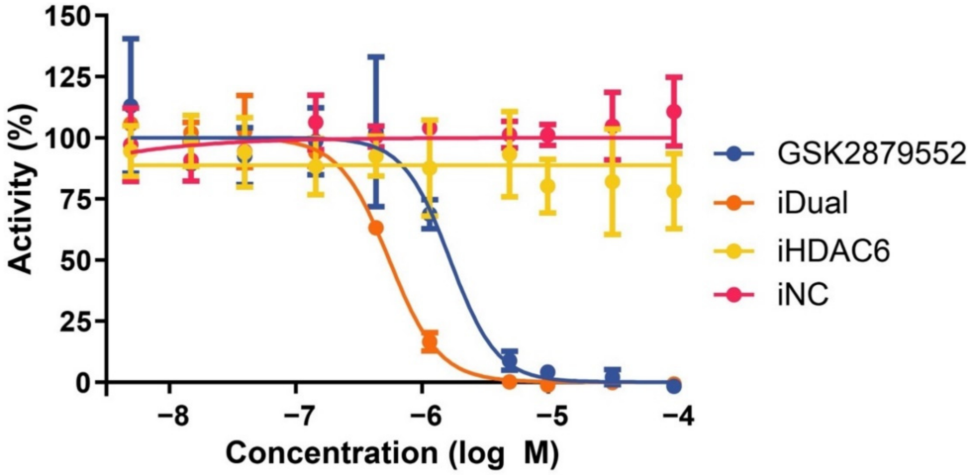

Figure S10. Dose-response curves (IC_{50}) of inhibition of LSD1 detecting the level of demethylation of K4me1 peptide substrate using MALDI-TOF screening assay. Conditions: $H3_{1-21}$K4me1 (5.0 μM) against LSD1 (0.15 μM), incubated for 10 min and quenched with formic acid (2%). N: 2-3 technical replicates, error given as St Dev.

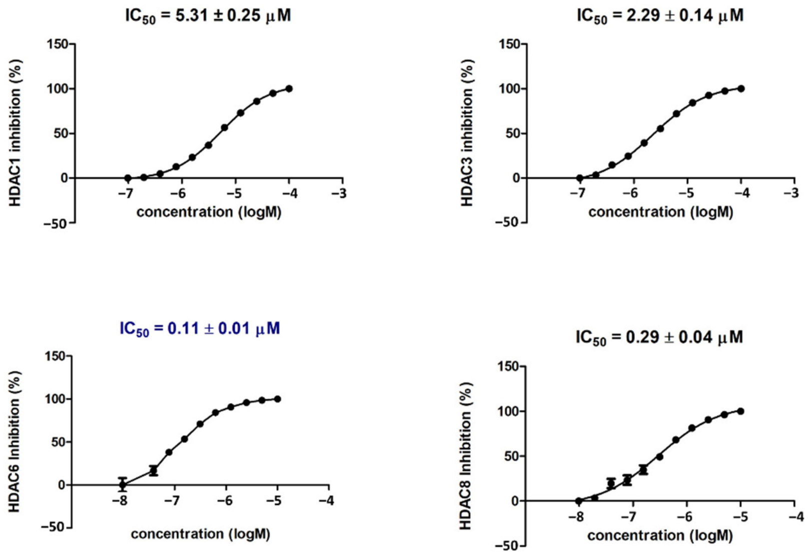

Figure S11. Dose-response curves for iDual in HDAC isoform enzyme assays.

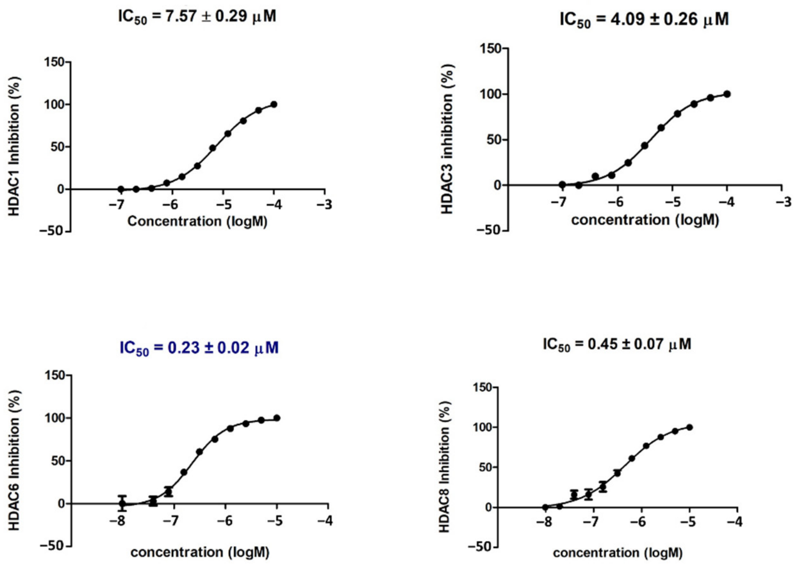

Figure S12. Dose-response curves for iHDAC6 in HDAC isozyme enzyme assays. Values were extracted from fitting dose-response curves to the data points using GraphPad software. Trichostatin A was employed as the positive control, and gave the following IC_{50} values (nM): HDAC1 1.01 ± 0.23; HDAC3 1.91 ± 0.25; HDAC6 2.15 ± 0.87; HDAC8 296.6 ± 80.7.

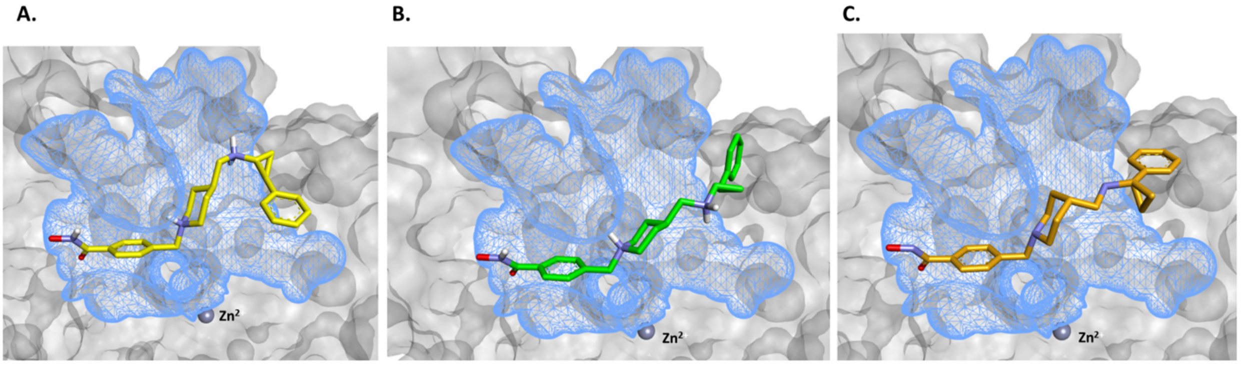

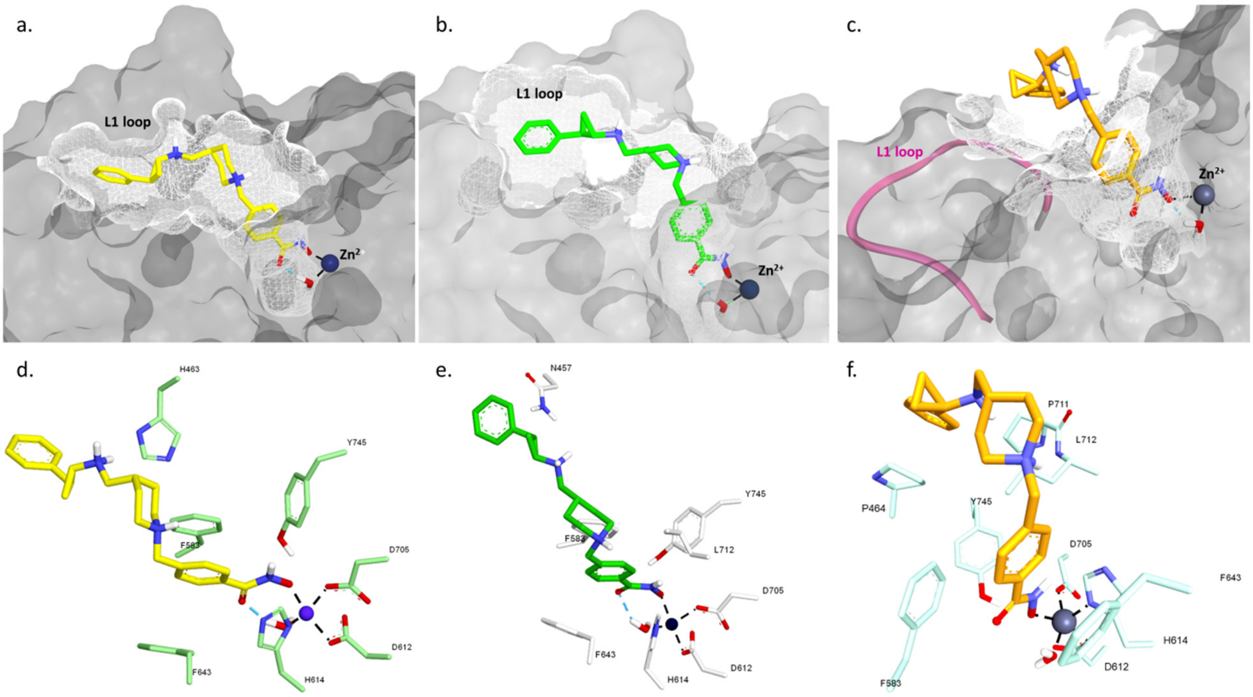

Figure S13. Predicted docking poses of iDual and iHDAC6 in the HDAC1 and HDAC6 active site. (**A**) Predicted docking poses of iDual (1*R*,2*S*, yellow), (**B**) iDual (1*S*,2*R*, green) and (**C**) iHDAC6 (orange) with the HDAC1 crystal structure (PDB: 5ICN) (upper panel). Docking poses of inhibitors bound to the second catalytic domain of HDAC6: (**a**) iDual (1*R*,2*S*, yellow), (**b**) iDual (1*S*,2*R*, green) and (**c**) iHDAC6 (orange). The amino acid residues interacting with the inhibitors are depicted underneath in (**d**), (**e**) and (**f**). Interactions between the hydroxamic acid and the zinc ion are shown as black dashed lines (lower panel).

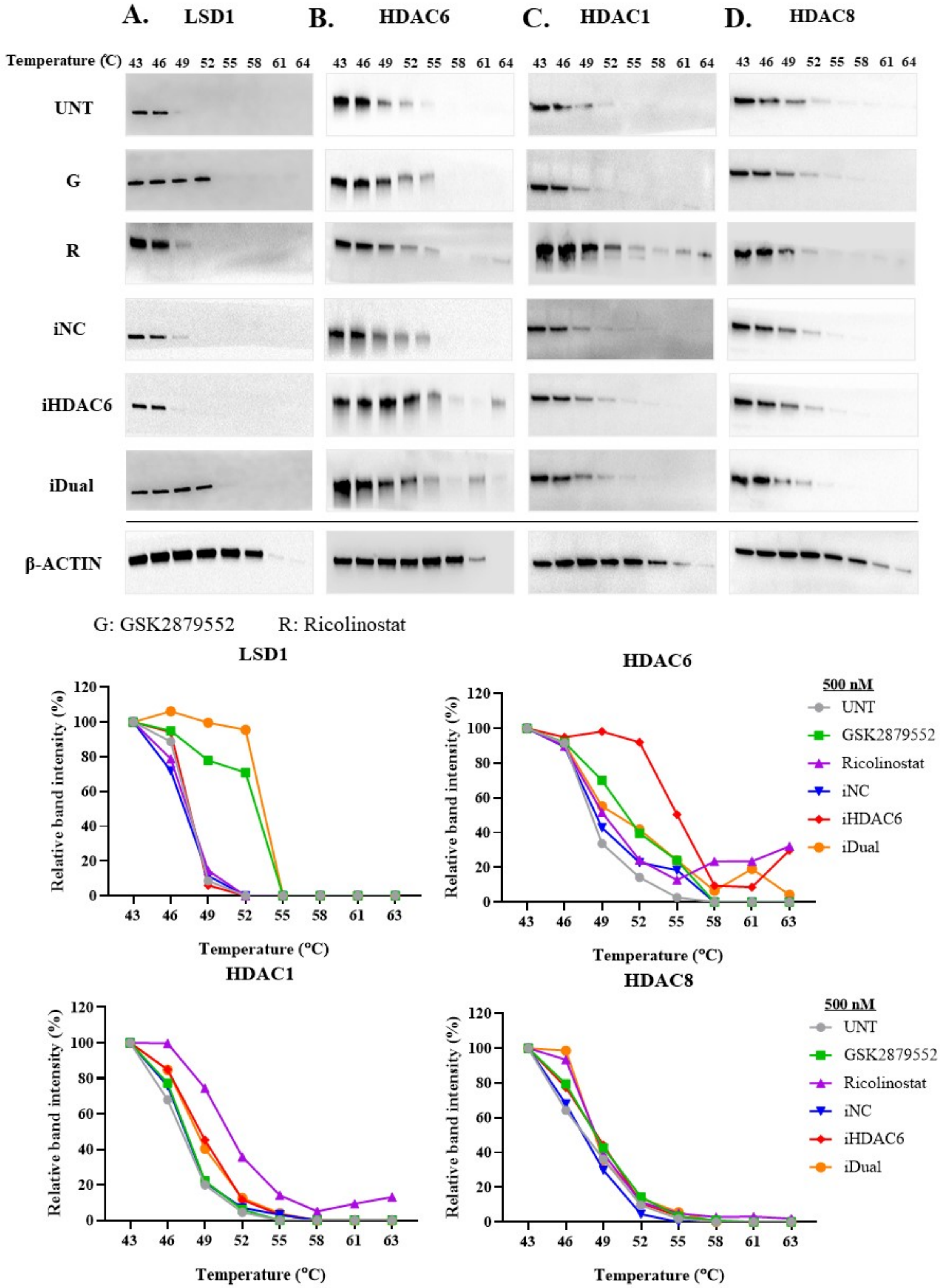

Figure S14. CETSA of LSD1 and HDAC isozymes in THP-1 cells treated with DMSO (untreated, UNT) or inhibitors and negative control **4** (500 nM, 1 hour). β-Actin is included as a loading control. Relative band intensities were quantified using Image J software and indicated graphically for (**A**)

LSD1, (**B**) HDAC6, (**C**) HDAC1 and (**D**) HDAC8. Full Western Blot Images can be found in Figure S31.

A.

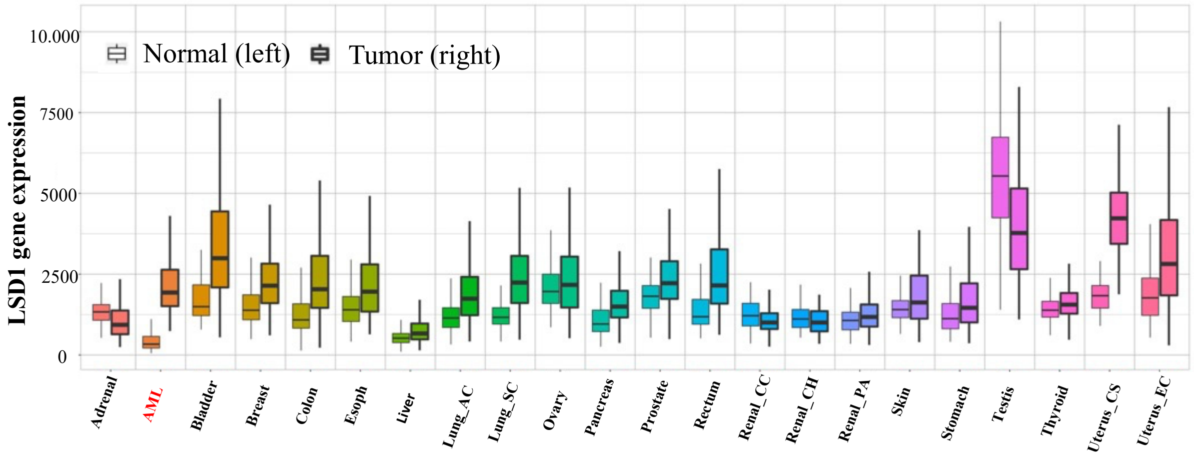

B.

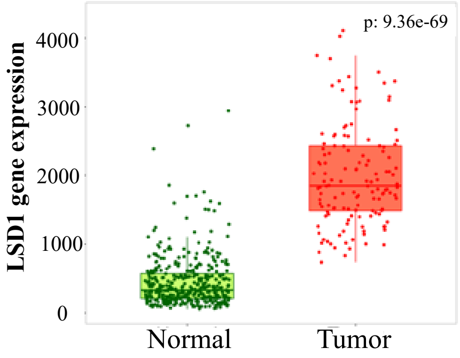

Figure S15. LSD1 (KDM1A) differential gene expression analysis of normal and tumor tissues (**A**) LSD1 expression on a different tissue panel compared with normal and tumor samples. (**B**) The normal and tumor analysis provides detailed analysis for LSD1 gene in a selected AML tissue type using RNA-Seq based data. Retrieved from https://tnmplot.com/analysis/, (accessed on 20 October 2022).

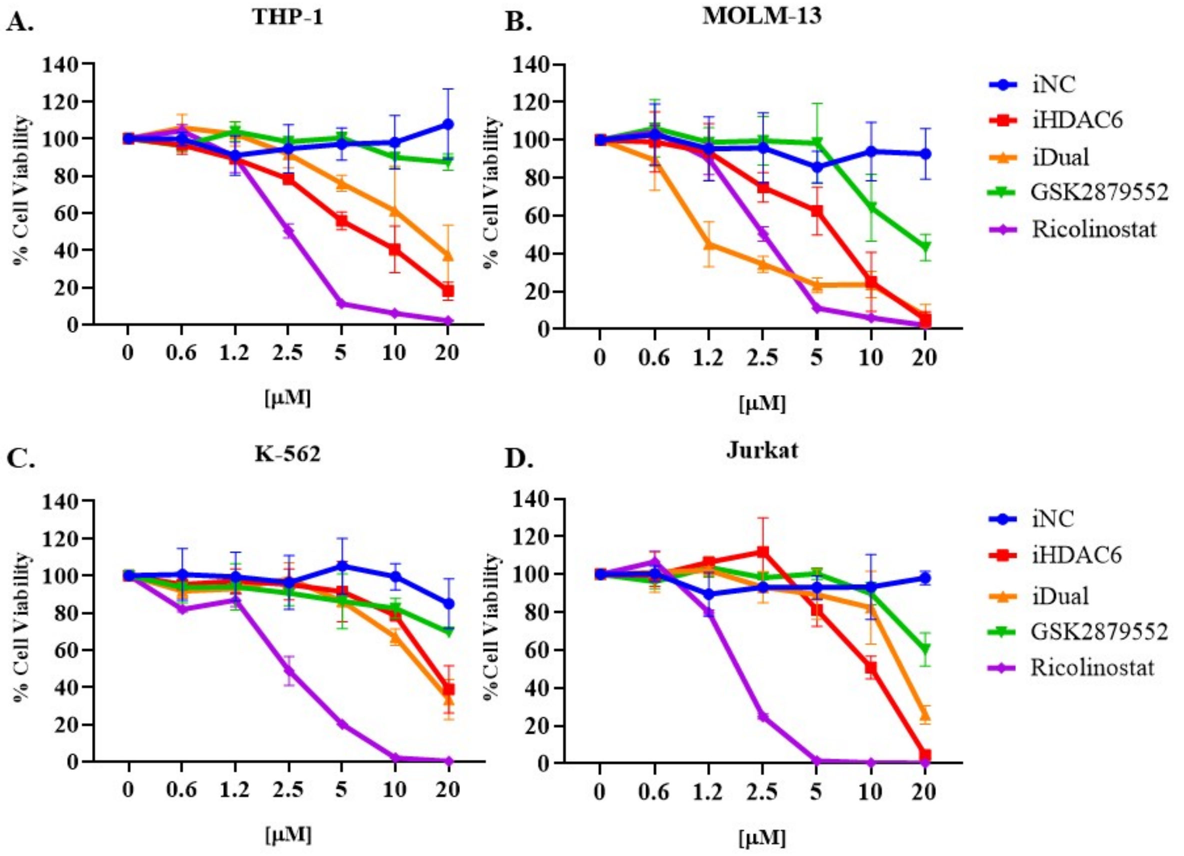

Figure S16. Cell viability dose-response curves of AML cell lines treated with iNC, iHDAC6, iDual, GSK2879552 and ricolinostat: (**A**) THP-1, (**B**) MOLM-13, (**C**) K-562 and (**D**) Jurkat cells. Cell viability was measured using ATP-dependent CTG assay and cell viability normalized to vehicle treated cells. Data represented as the average of ± standard deviation from three biological repeats.

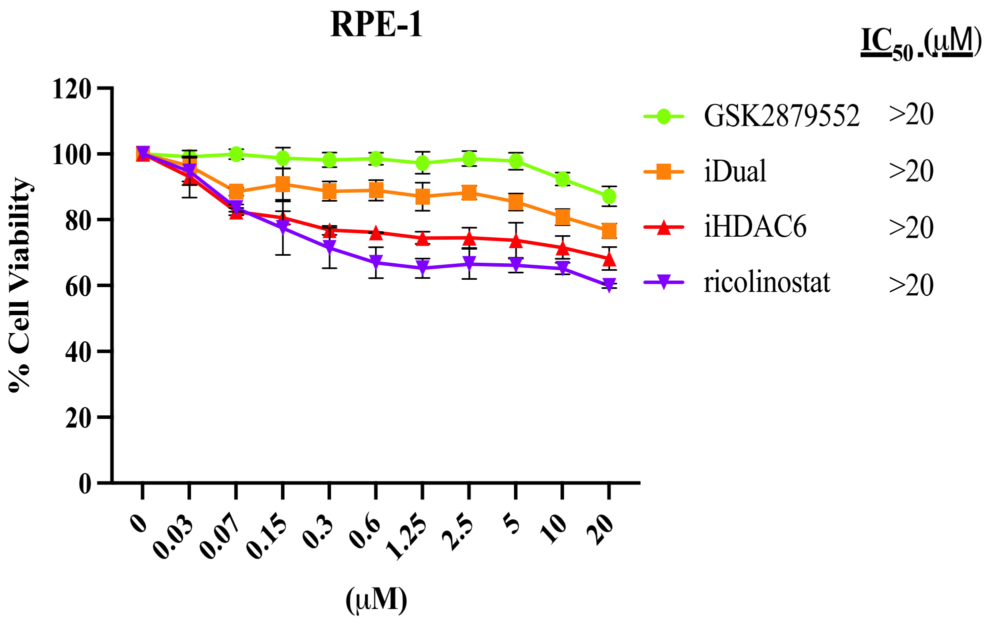

Figure S17. Cell viability dose-response curves of normal RPE-1 cell lines treated with GSK2879552, iHDAC6, iDual, and ricolinostat for 120 h. Cell viability was measured using ATP-dependent CTG assay and cell viability normalized to vehicle treated cells. Data represented as the average of ± standard deviation from three biological repeats.

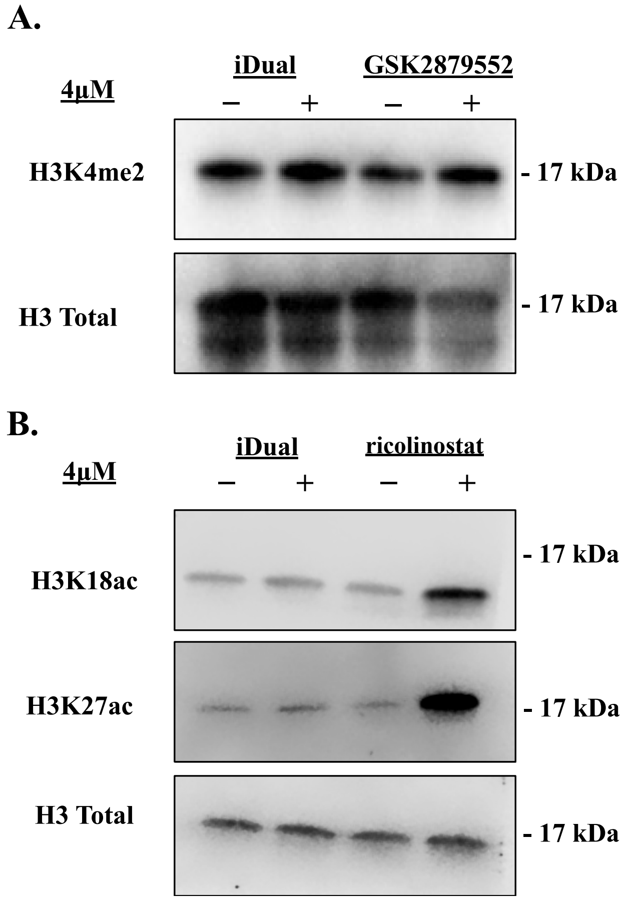

Figure S18. Western blots of methylation and acetylation levels of LSD1 and HDAC substrates in THP-1 cells incubated with 4 µM of compounds for 24 h. Isolated histones were run on SDS-PAGE gels and blotted for indicated antibodies for: (**A**) H3K4me2 (**B**) H3K18ac/H3K27ac. Full Western Blot images can be found in Figure S32.

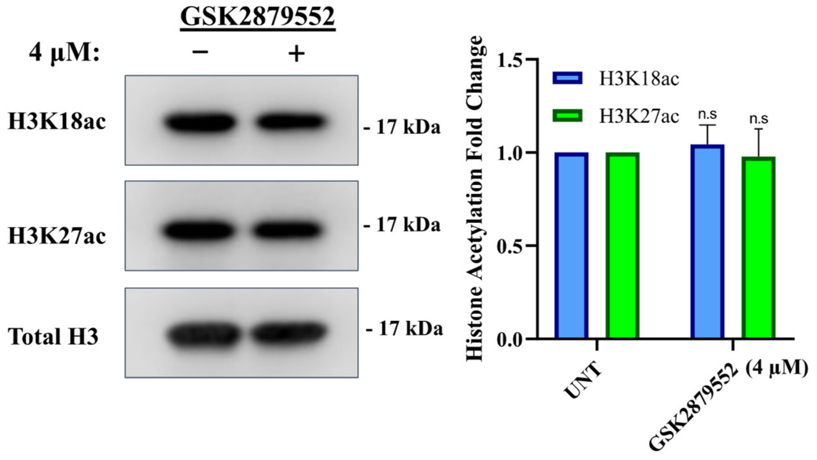

Figure S19. THP-1 cells were treated with GSK2879552 (4 µM, 24 h) and isolated histones (2 µg) were run on SDS-PAGE and blotted for indicated antibodies. The fold changes were quantified from band intensities via Image J software. Data represented as the average of ± standard deviation from two biological repeats (n.s: not significant). Full Western Blot images can be found in Figure S33.

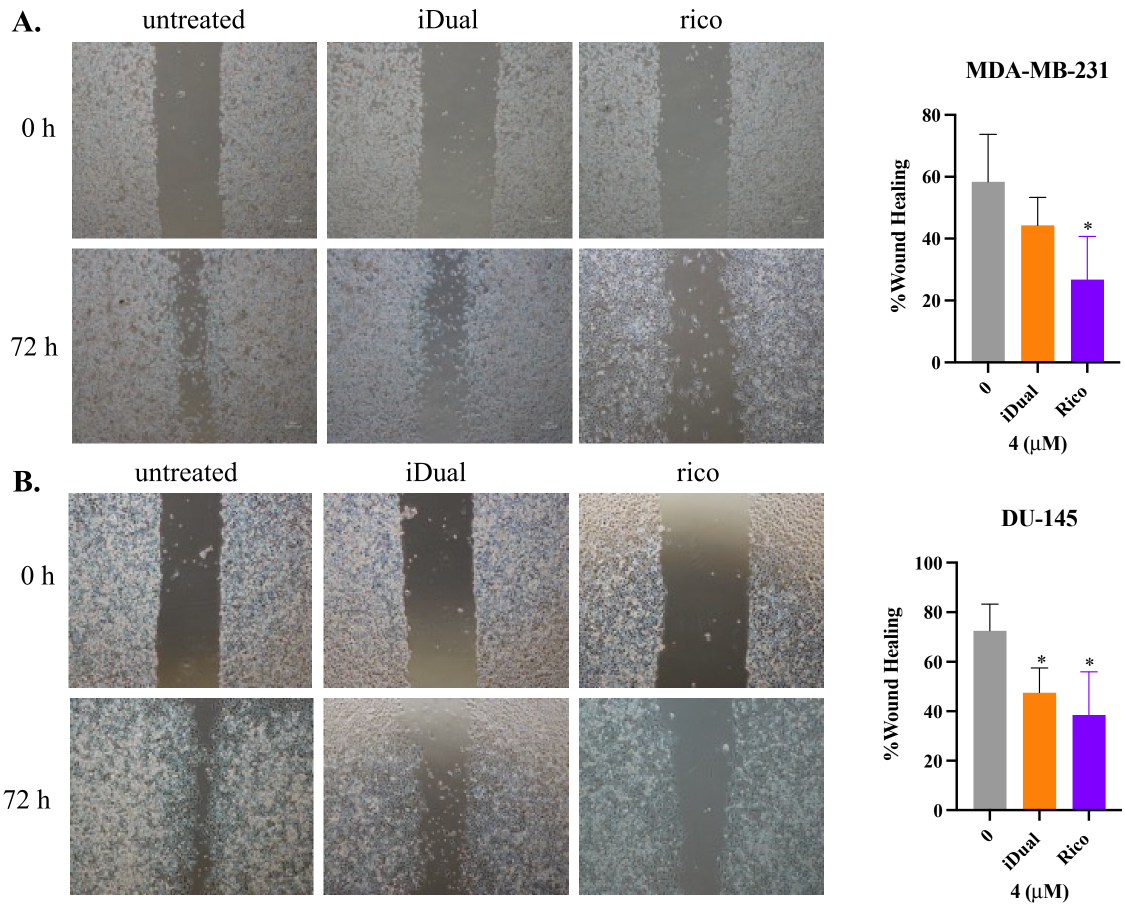

Figure S20. Wound healing assay with iDual and ricolinostat. (**A**) MDA-MB-231 and (**B**) DU-145 cells were seeded on 6 well plates at 80–100% confluency, following treatment with 4 μM of iDual and ricolinostat for 72 h cells grown in relative medium with (1%) FBS conditions. Wound healing is photographed with Nikon Eclipse TS 100 microscope. Wound/healed area is quantified using Image J software. (* $p < 0.05$)

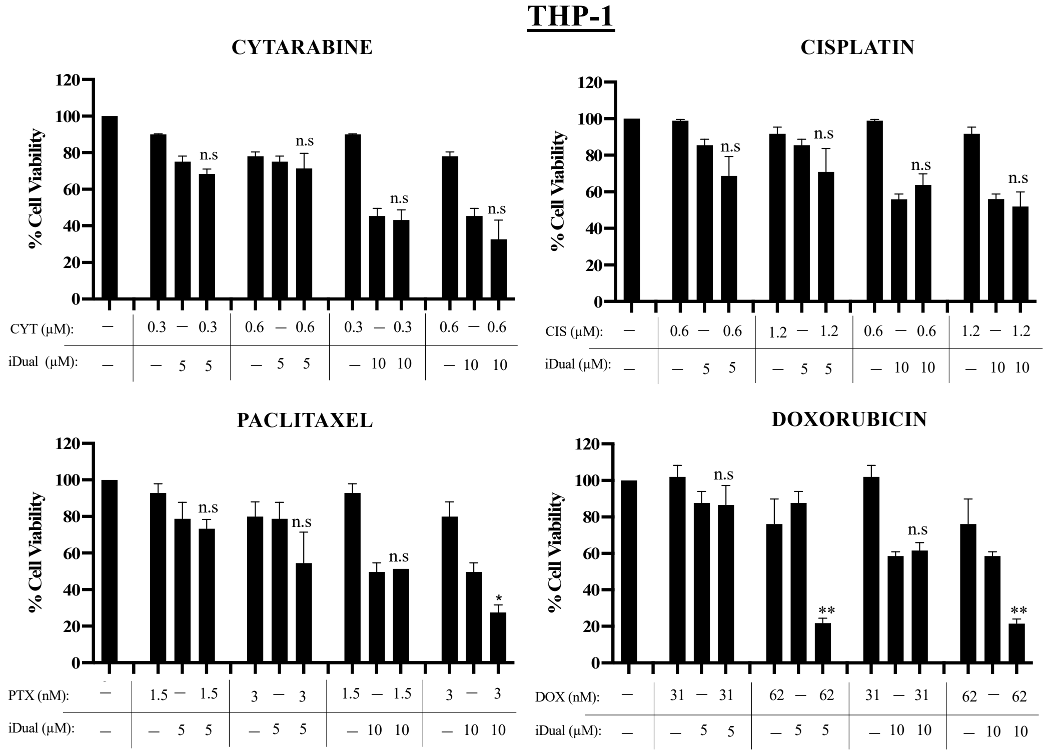

Figure S21. Cell viability graphs of THP-1 cells following treatment with iDual or four different chemotherapy drugs using two different dose combinations. Cells were treated with epidrug alone for 72 h, then co-treated with the antileukemic drug for 48 h. Cell viability was measured using ATP-dependent CTG assay, and cell viability was normalized to vehicle treated cells. Data represented as the average of ± standard deviation from the mean of two biological repeats. (* $p < 0.05$, ** $p < 0.01$, n.s: not significant)

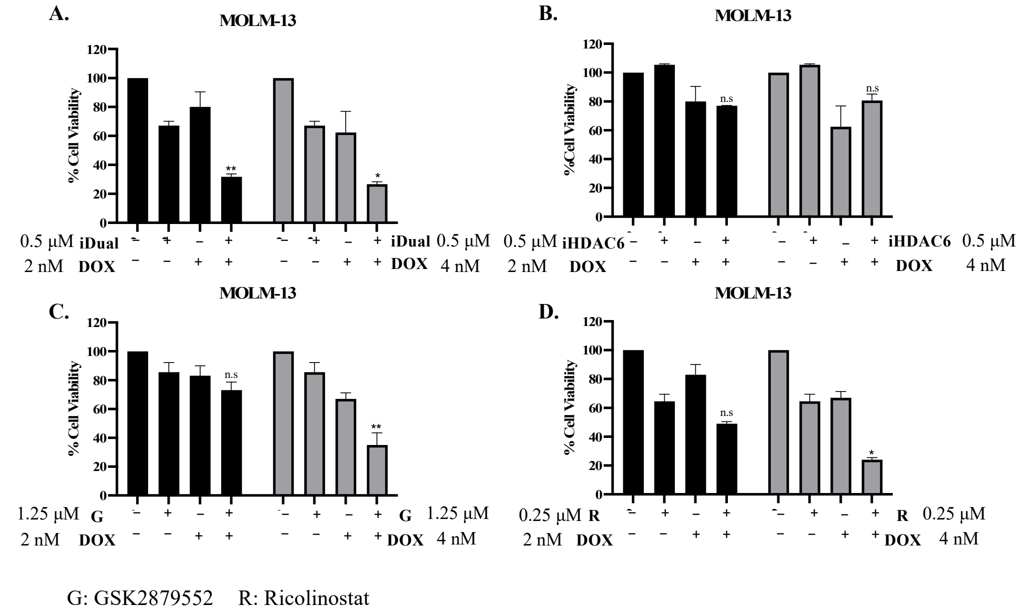

Figure S22. Drug combination studies using LSD1/HDAC inhibitors with doxorubicin on MOLM-13 cells. MOLM-13 cells were treated with (**A**) iDual, (**B**) iHDAC6, (**C**) GSK2879552 and (**D**) ricolinostat in combination with doxorubicin using two different doses. Cells were treated with epidrugs alone for 72 h, then co-treated with doxorubicin for 48 h. Cell viability was measured using ATP-dependent CTG assay, and cell viability was normalized to mock treated cells. Data

represented as the average of ± standard deviation from the mean of two biological repeats. (* $p < 0.05$, ** $p < 0.01$, n.s: not significant).

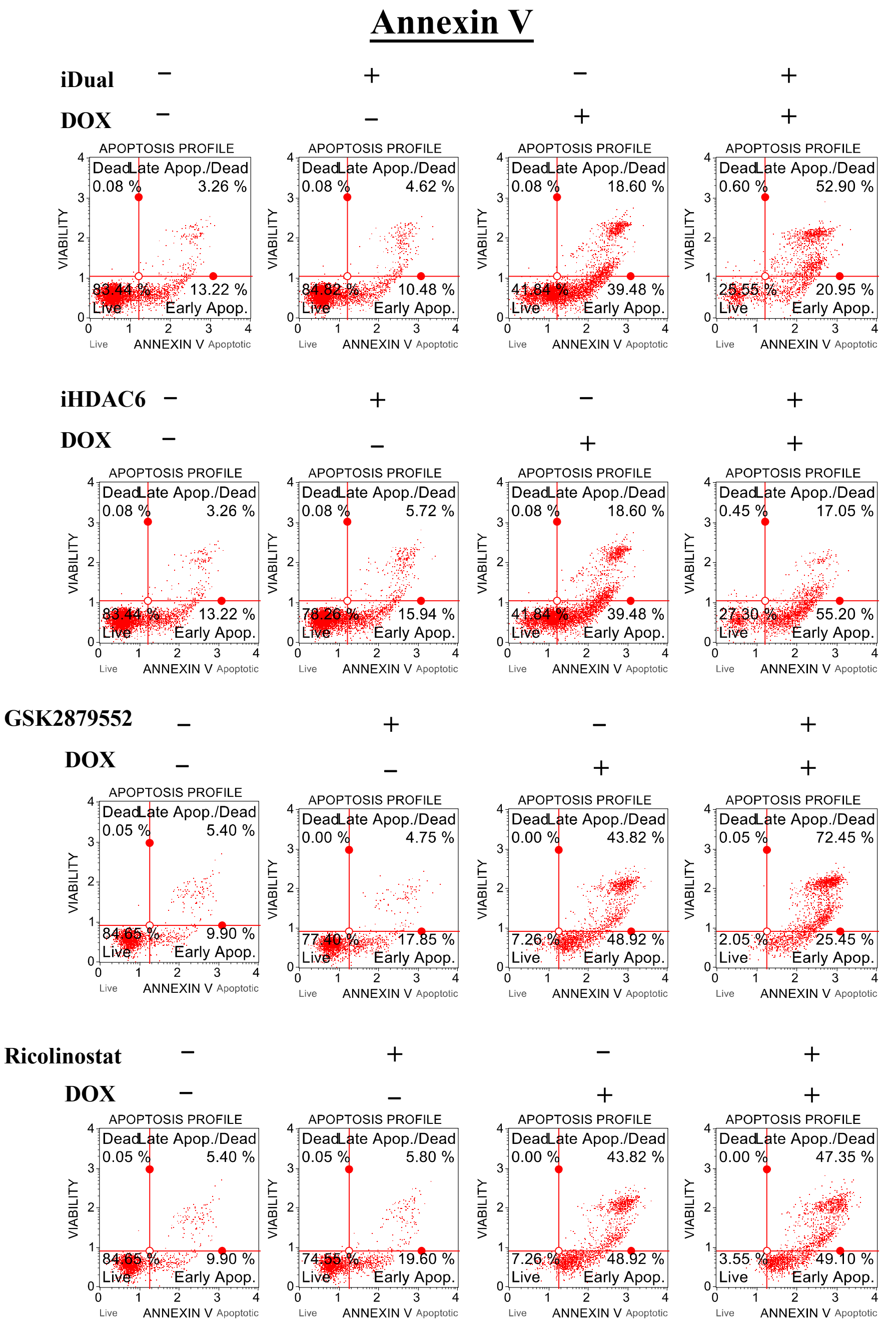

Figure S23. Flow cytometric analysis of Annexin V positivity after combination treatments. THP-1 cells were pretreated with indicated inhibitors for 72 h, followed by co-treatment with doxorubicin for 48 h. Cells were analysed with Muse Cell Analyzer for Annexin V and 7-AAD staining. Representative images showing the data from two biological repeat.

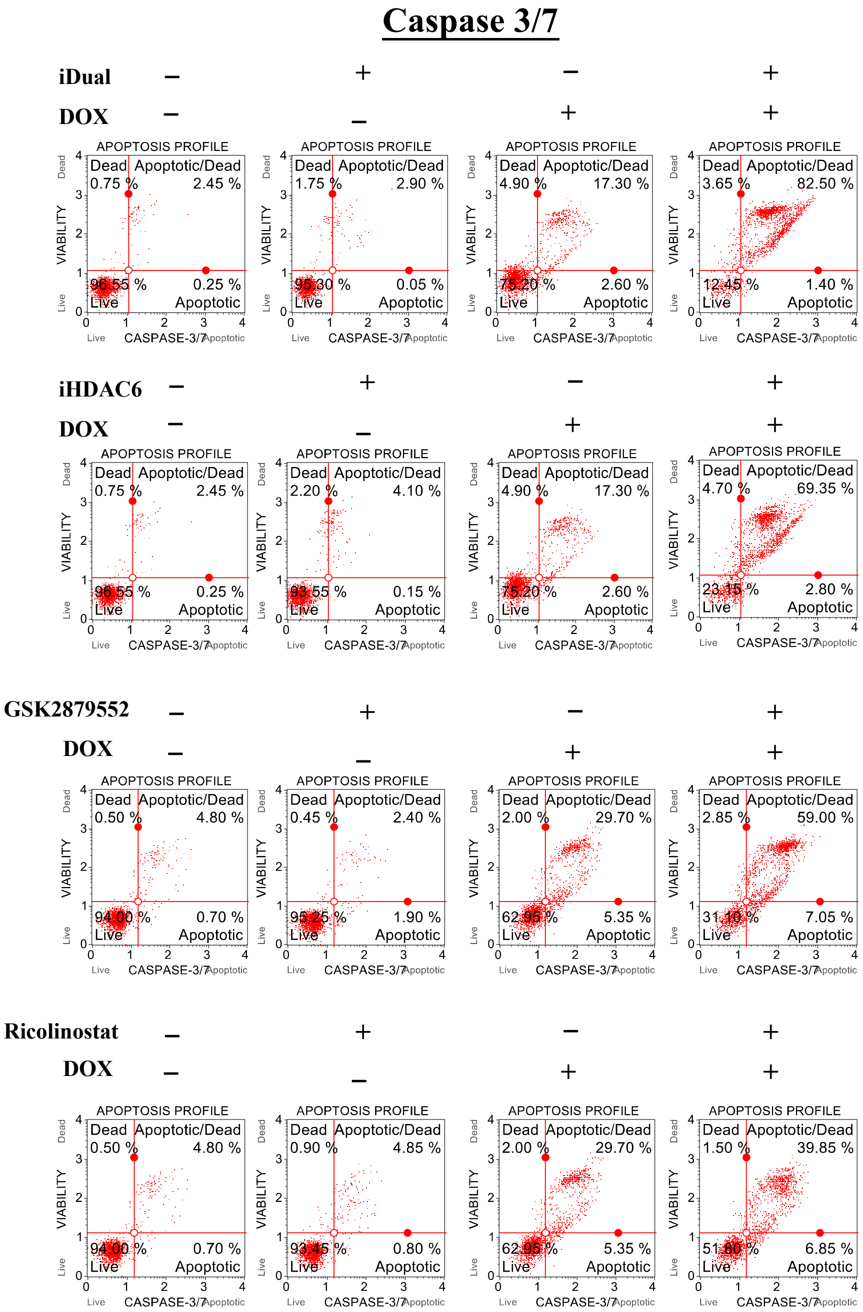

Figure S24. Flow cytometric analysis of Caspase 3/7 activation after combination treatments. THP-1 cells were pretreated with indicated inhibitors for 72 h, followed by co-treatment with doxorubicin for 48 h. Cells were analysed with Muse Cell Analyzer for Caspase 3/7 and 7-AAD staining. Representative images showing the data from two biological repeats.

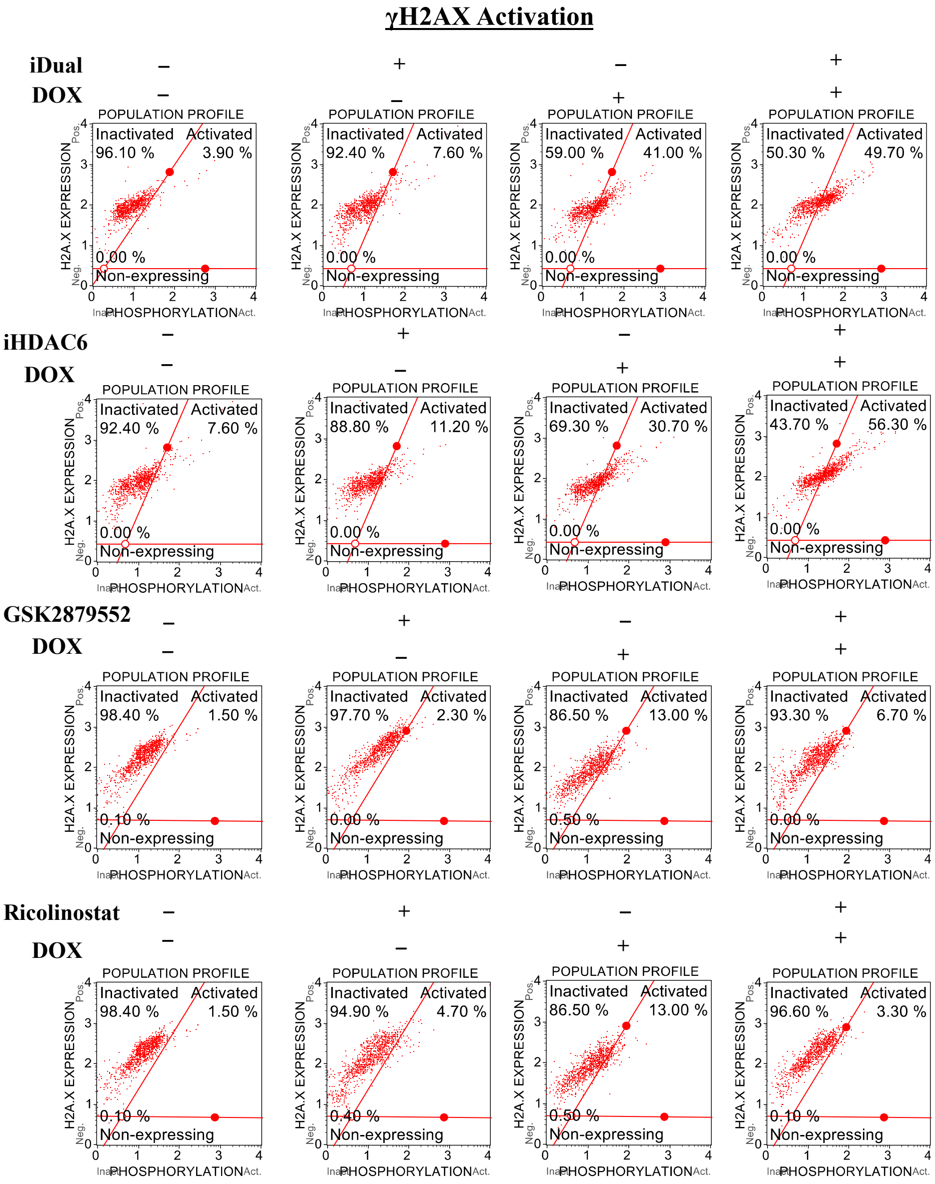

Figure S25. Flow cytometric analysis of double strand breaks after combination treatments. THP-1 cells were pretreated with indicated inhibitors for 72 h, followed by co-treatment with doxorubicin for 6 h. Cells were analysed with Muse Cell Analyzer for H2AX activation. Images represent as the average from two biological repeats. The inactivated quadrant indicates the cells expressing H2AX without phosphorylation and the activated quadrant indicates the γH2AX phosphorylated cells.

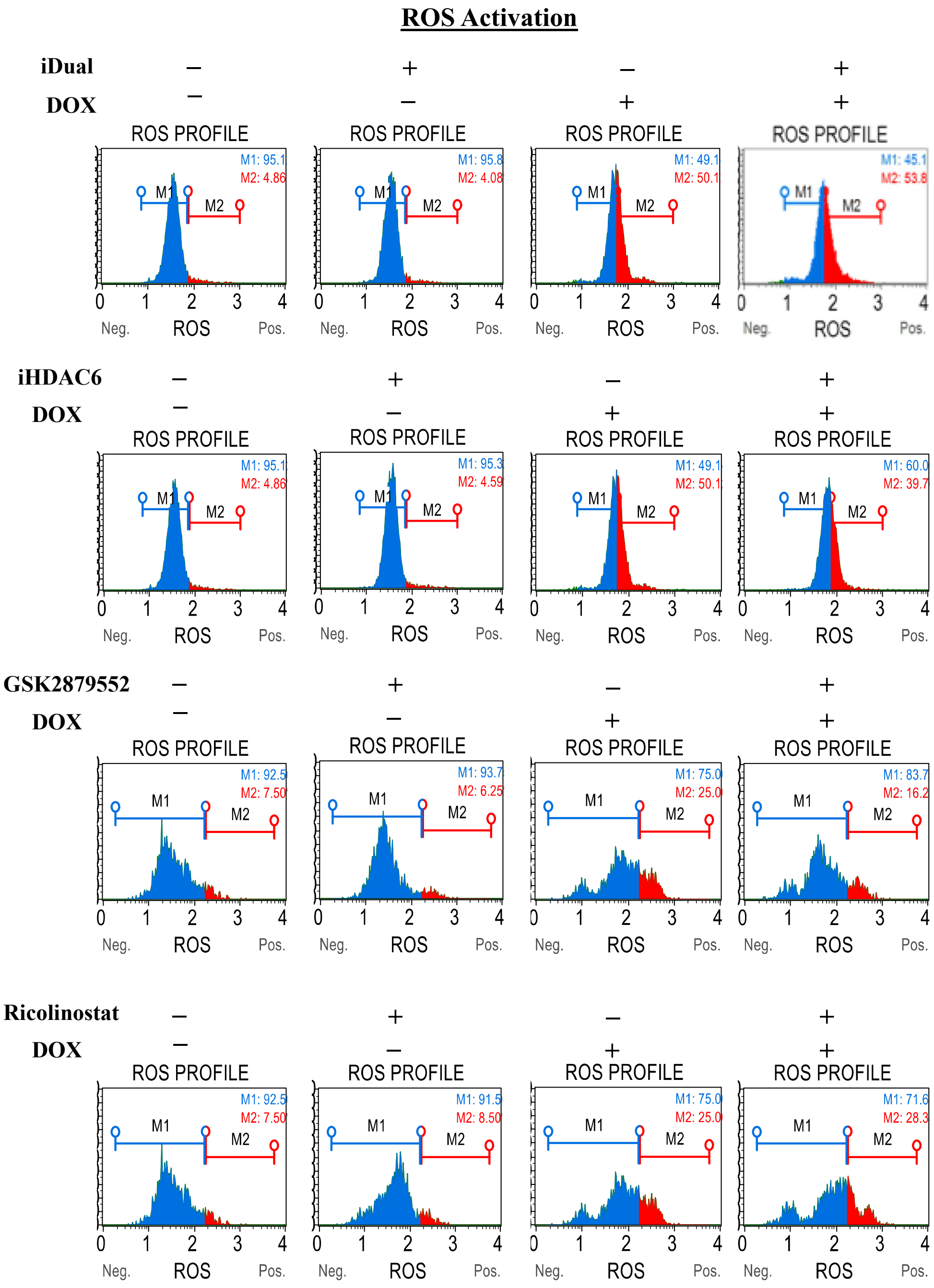

Figure S26. Flow cytometric analysis of oxidative stress after combination treatments. THP-1 cells were pretreated with indicated inhibitors for 72 h, followed by co-treatment with doxorubicin for 24 h. Generation of oxidative stress in THP-1 cells was assessed by Muse cell analyzer. Data represented as the average of ± standard deviation from two biological repeats.

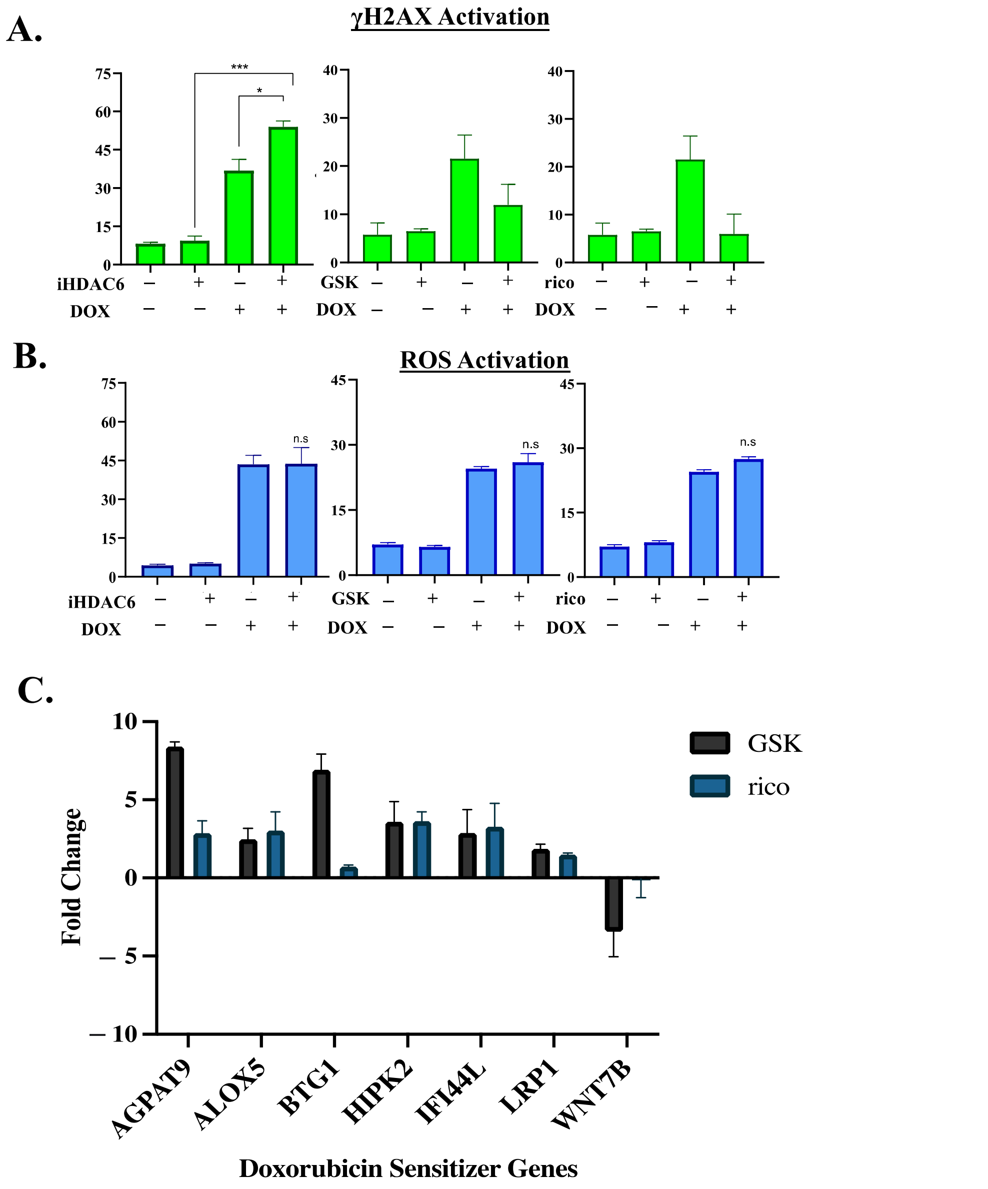

Figure S27. Flow cytometric analysis of other drugs and gene expression of doxorubicin sensitizer genes on single targeted drugs. (**A, B**) Flow cytometric graphs of DNA breaks and oxidative stress after combination treatments. THP-1 cells were pretreated with indicated inhibitors for 72 h, followed by co-treatment with doxorubicin for 24 h. (**C**) Doxorubicin sensitizer gene expression analysis after single targeted LSD1 or HDAC6 inhibitor Data represented as the average of ± standard deviation from two biological repeats. (* $p < 0.05$, *** $p < 0.001$, n.s: not significant)

Table S1. Primer sequences.

Gene	Forward Sequence (5′–3′)	Reverse Sequence (5′–3′)
CD11b	TGTCAAGGGCACCCAGATCG	AAGGGGCACACGGACACC
CD86	GGACTAGCACAGACACACGGAT	AGCAGCACCAGAGAGCAGGAAG
GAPDH	CTGACTTCAACAGCGACACC	GTTGTCATACCAGGAAATGAGC
P21	TCGTCCAGCGACCTTCCTCA	CAGCCCCATATGAGCCCACG
OGG1	CAGCCCCATATGAGCCCACG	GACATCATCAAGCTGGATGAG
MUTYH	CATATCAAGTATATGGGCTGGC	CTTTTGGAACCCATACAGGTC
GADD45A	GCCCAATTAGTGTCGTGCG	TTCTGCACTCACTCACAGGC
XPA	CTTGTGATAACTGCAGAGATGC	CAATAAATTTAAGAGGTGGCTCTC
ERCC1	CCAGGTGGATGTGAAAGATCC	CTTGTAGGTCTCCAGGTACC
ERCC3	TGCTGTACACCATGAACCCC	CCCTGAGACGTAGGTCCGTA
RAD51	TTGAAGCAAATGCAGATACTTCAG	GGCATTTATGCCACACTGCTC

RAD52	AGAAGGTGTGCTACATTGAGG	GATATGAACCATCCTTCAGCTG
ATM	TTCCATCGCATGTGATTAAAGCA	AATTTTCTGATAGGAATCAGGGC
ATR	ACCAATTGTGGAGGAGATTTCC	ACTTCTGAGAACTCTTGTATCTG
XRCC5	TATTTCAGTGTCTGCTGCACAG	GGGAGGATTCAGCATATTCCA
LIG4	GTAGTTTCTTGTTTAGCTGAGGG	TGCAACACGACTATGATCTTCC
MSH2	TTGATGGCCAGAGACAGGTT	CCATGTCTCCAGCAGTCTCT
MSH3	CCATCATGGCTCAGATTGGC	ATCATGAGTGCTCGTCCCTC
MSH6	CTACTTCCAGCAACCCCAGA	AGAACCTCATGTCCCTGCTC
ABCA1	CCTTGCCAGCAAGACGAAAC	CCTTTGCCATCCATCCCACT
ABCA2	AAGGAAGTCTCCTTCTACACAGC	AAACAGGTTGCCTTCCTCCAC
ABCA3	CAGCTGGGCGAAGGTTTTC	GAGATCTGGCTCACGGAGTAGTC
ABCA4	GCTAAACAGCAGACTGAAAGTCATG	AAAGATCAGTCCTGGGCTTGTC
ABCA7	GTTCAGAAAGGGGCAGGGAG	GGAGCTGGACCGGCTGT
ABCA10	TCCTCTTTAATGGCGTATAAGTTACCT	TCTGTTTCATCGCCTCTAACTTGA
ABCB1	ACAGAGGGGATGGTCAGTGT	TCACGGCCATAGCGAATGTT
ABCB2	TAGCTCTAGGTGTCCCGCT	GTCGGCGAGAAGTAGCAGTA
ABCB3	GCATGGAATATACACAGATGTAGGG	CACTGTGCGATCCCCACG
ABCB5	GCAGAAGAACAGCCAAAACTGA	CAGGCTCCATTGACCAGTGAT
ABCB6	GTCTTGGAACAGCCCACAGT	CCCAGAGACCCAGGACAAAC
ABCB9	CGCAGTCTTCAATGAGCAGC	TGCTGAAGGCCAGGTTGTAG
ABCC1	AGGTGGACCTGTTTCGTGAC	CCTGTGATCCACCAGAAGGT
ABCC2	GCAGCGATTTCTGAAACACA	CAACAGCCACAATGTTGGTC
ABCC3	ACCCAGTTTGATACCTGCACTGT	GGACCCTGGTGTAGTCCATGA
ABCC5	ATTTGGACCCCTTCAACCAGTAC	GGTAGCTGAGCAATACATTCTTTCAT
ABCC8	CACCATCGCGCATCGA	GAGCAGCTTCTCTGGCTTATCG
ABCC11	GATGGTGCTTTGCAGCGAC	AGCATGTCATCAGTGAGCCC
ABCD3	GGCCTGCACGGTAAGAAAAG	CACAGCTCGCTCCTTTTTCC
ABCF3	GGTGGAAGCTGTAGGGGAAC	ATTTCCCTGGCTTTGTGGCT
ABCG1	TGTCTGATGGCCGCTTTCTC	CTGGACACCACCTCATCCAC
ABCG2	GCTTTCTACCTGCACGAAAACCAGTTGAG	ATGGCGTTGAGACCAG
AGPAT9	AGGAACTACACTGGTTGGGC	TCTGGGGTCTGTACTGCTTGT
ALOX5	ACGATGTCTACGTGTACGGC	AGTCGTACTGGCCGAAGTTG
BTG1	ATCTCCAAGTTTCTCCGCACC	CTTGCATGGCTTTTCTGGGA
HIPK2	TCCTTTGTGGAGACTGAAGACAC	TCGCTCCCTTCCAAATCTGTC
IFI44L	TTGGAACTGGACCCCATGAAG	AATGCAGGGCTGTAACGCT
LRP1	ATGGTGCAGACGAGAGCATC	CAGGTCGGGTACTCACACTC
WNT7B	CGTGTTTCTCTGCTTTGGCG	GAACTGGTACTGGCACTCGT

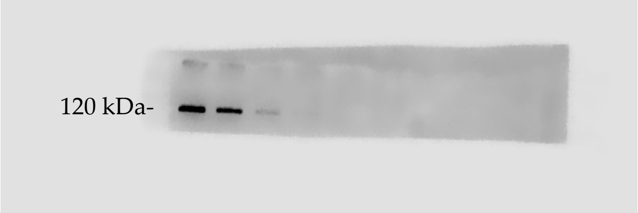

Figure 2B. Untreated-LSD1 blotting

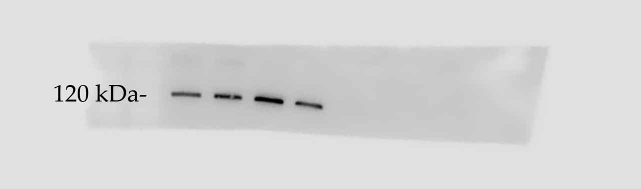

Figure 2B. iDual-LSD1 blotting

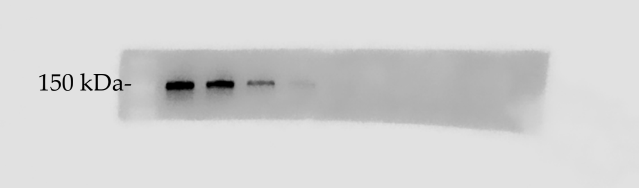

Figure 2B. Untreated-HDAC6 blotting

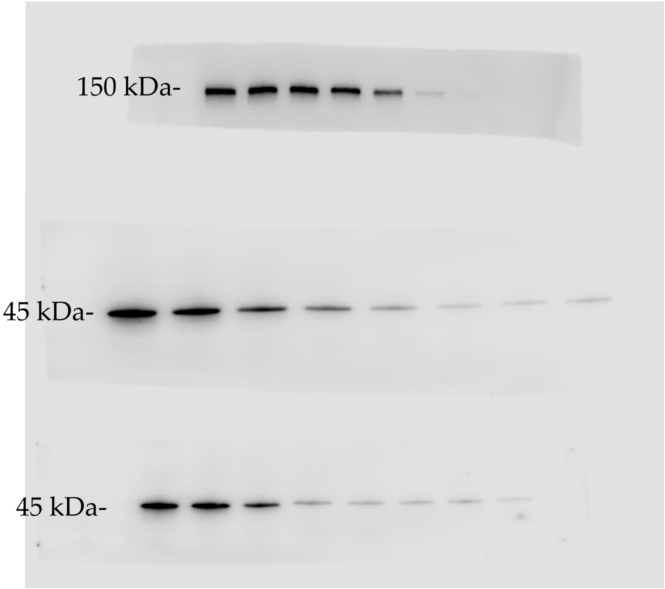

Figure 2B. iDual-HDAC6 blotting

Figure 2C. Untreated-HDAC8 blotting

Figure 2C (iDual-HDAC8 blotting)

Figure S28. Uncropped Western Blot images from Figure 2.

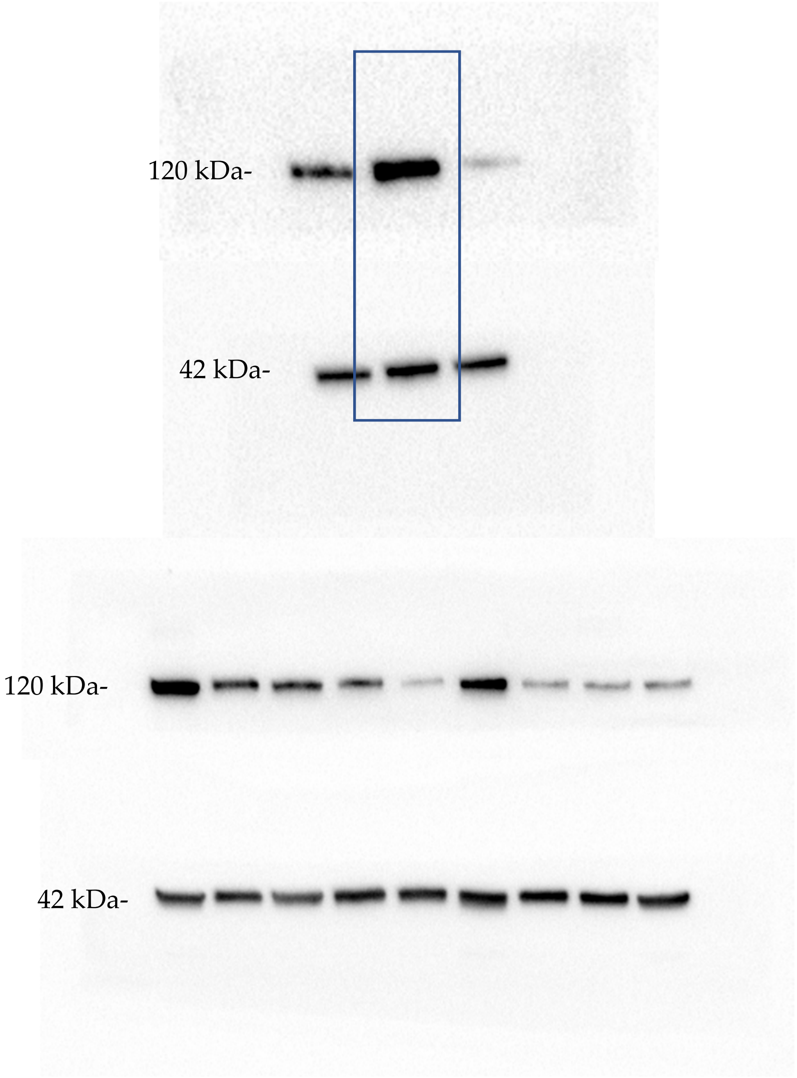

MOLM-13-LSD1 blotting

MOLM-13-Beta-actin blotting

THP-1 starting series-LSD1 blotting

THP-1 starting series-Beta actin blotting

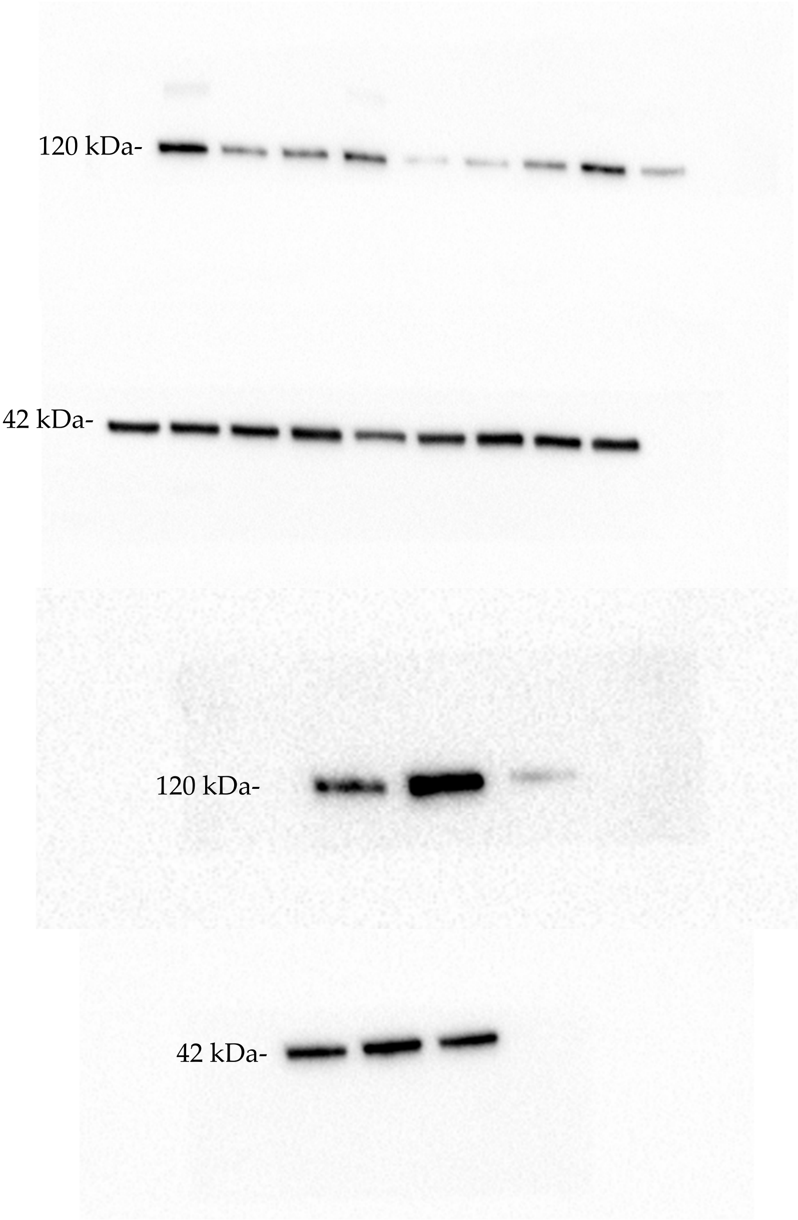

22Rv1 starting series-LSD1 blotting

22Rv1 starting series-Beta actin blotting

A549 starting series-LSD1 blotting

A549 starting series-Beta actin blotting

Figure S29: Uncropped Western Blot images from Figure 3A.

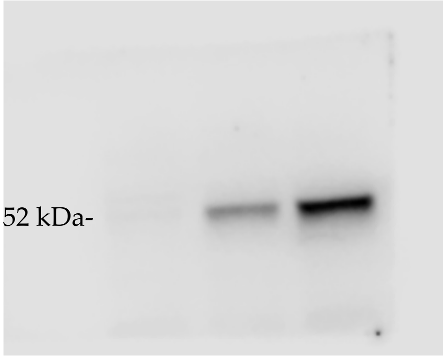

Figure 4C (K40ac-alfa TUBULIN) Gel1

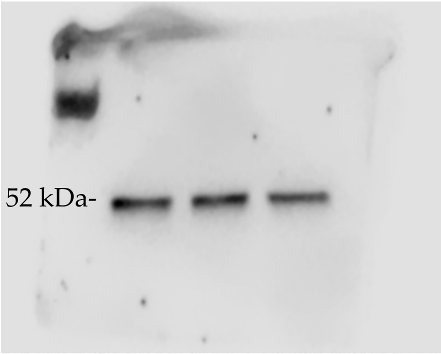

Figure 4C (alfa-TUBULIN) Gel 1

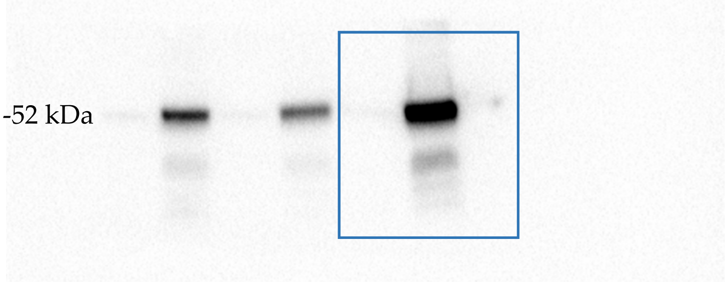

Figure 4C (K40ac-alfa TUBULIN) Gel 2

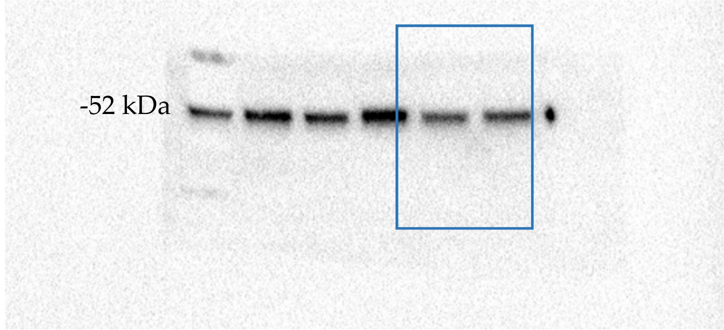

Figure 4C (alfa-TUBULIN) Gel 2

Figure S30. Uncropped Western Blots from Figure 4.

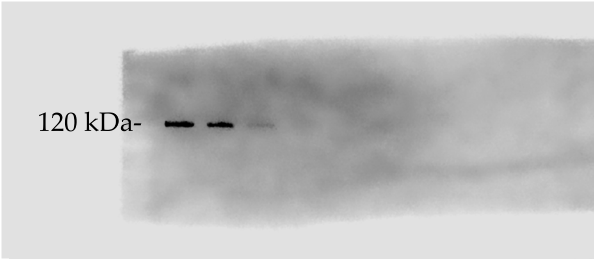

Figure S14A (UNT-LSD1 blotting)

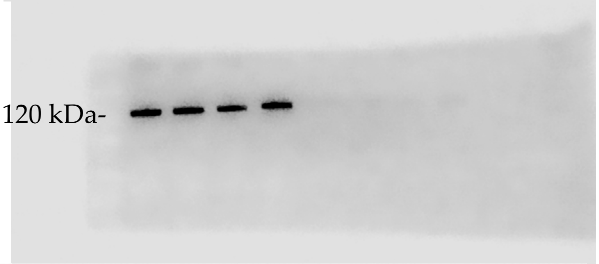

Figure S14A (GSK2879552-LSD1 blotting)

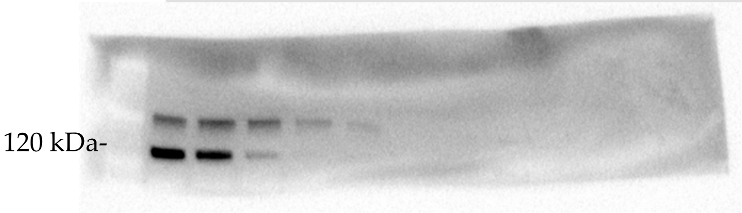

Figure S14A (Ricolinostat-LSD1 blotting)

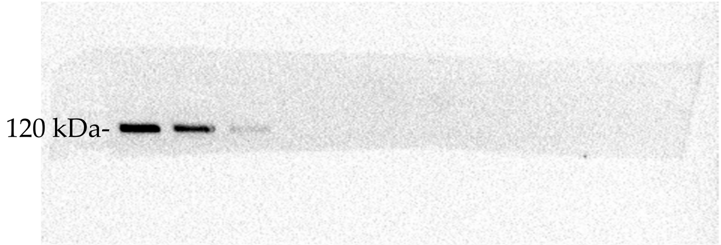

Figure S14A (iNC-LSD1 blotting)

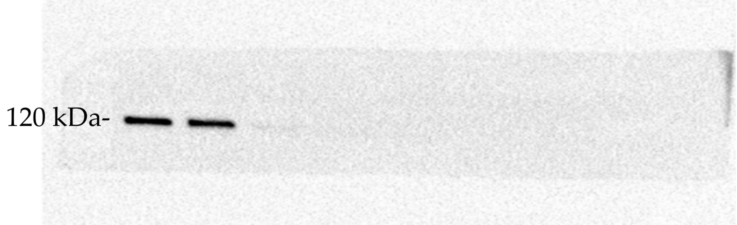

Figure S14A (iHDAC6-LSD1 blotting)

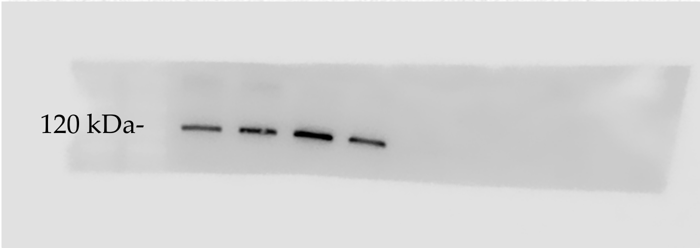

Figure S14A (iDual-LSD1 blotting)

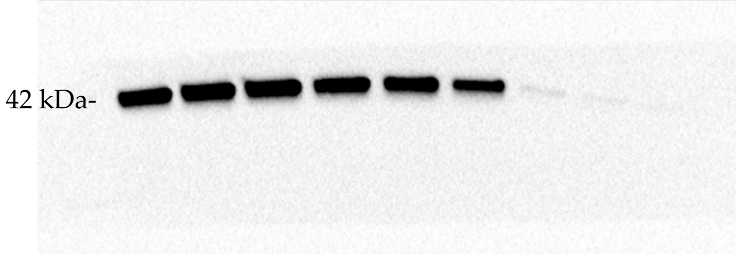

Figure S14A (Beta-actin blotting)

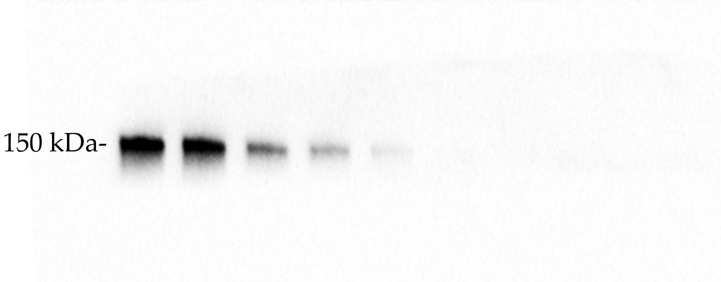

Figure S14B (Untreated-HDAC6 blotting)

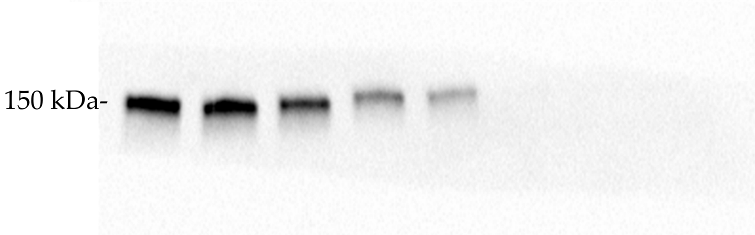

Figure S14B (GSK2879552-HDAC6 blotting)

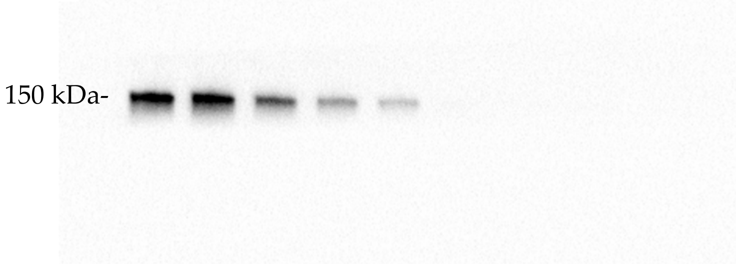

Figure S14B (iNC-HDAC6 blotting)

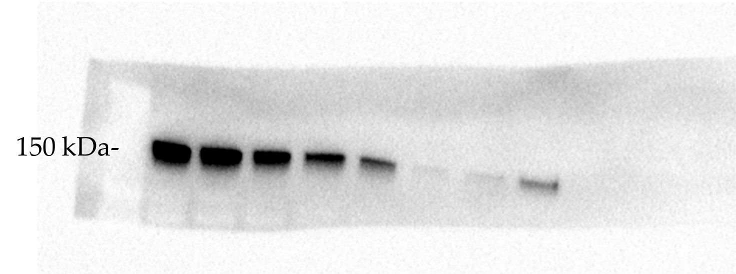

Figure S14B (Ricolinostat-HDAC6 blotting)

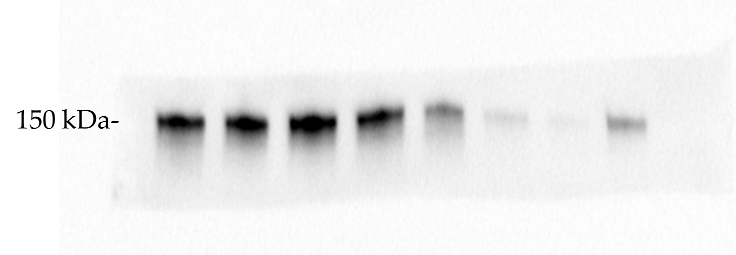

Figure S14B (iHDAC6-HDAC6 blotting)

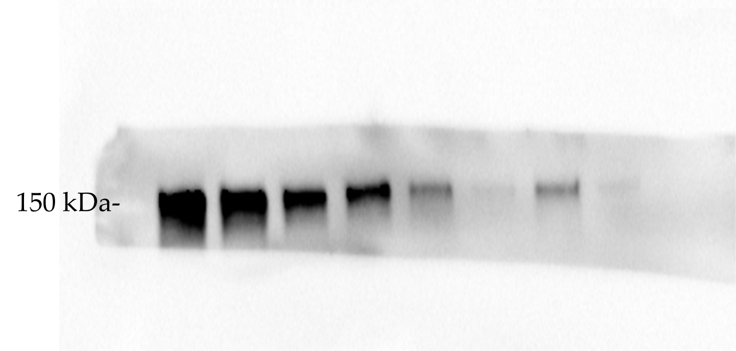

Figure S14B (iDual-HDAC6 blotting)

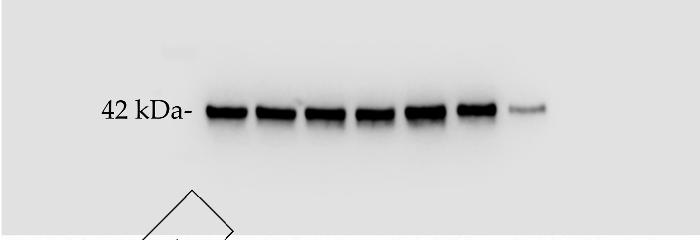

Figure S14B (Beta actin blotting)

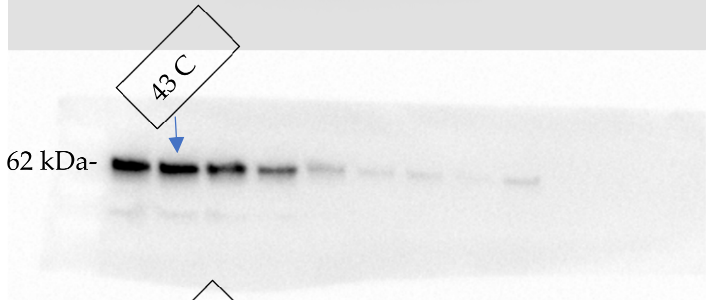

Figure S14C (UNT-HDAC1 blotting)

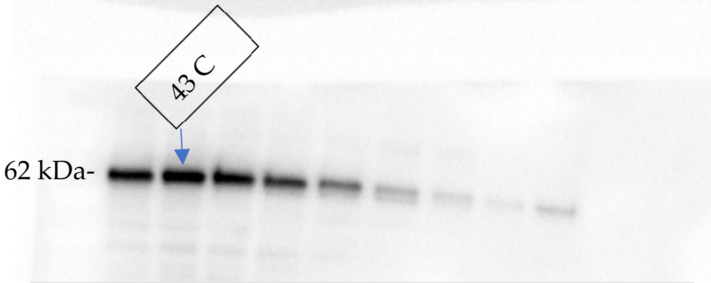

Figure S14C (GSK2879552-HDAC1 blotting)

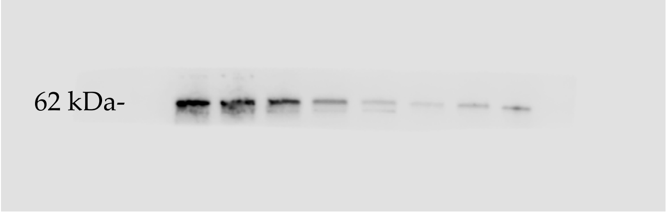

Figure S14C (Ricolinostat-HDAC1 blotting)

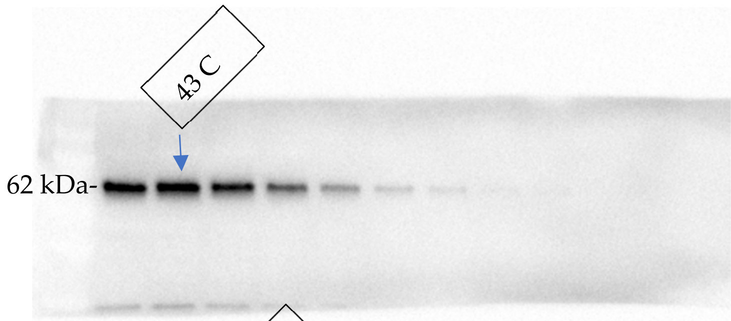

Figure S14C (iNC-HDAC1 blotting)

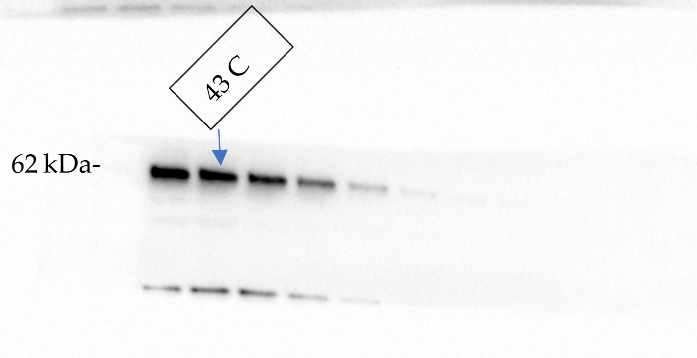

Figure S14C (iHDAC6-HDAC1 blotting)

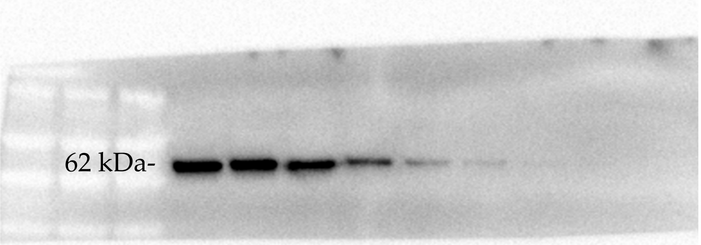

Figure S14C (iDual-HDAC1 blotting)

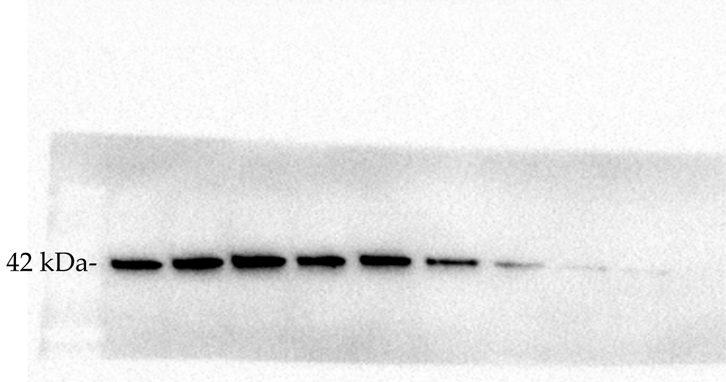

Figure S14C (Beta actin blotting)

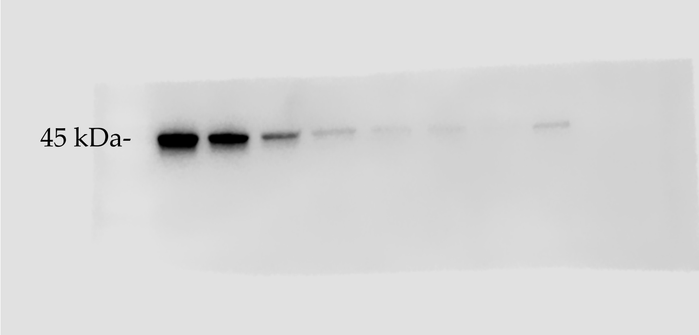

Figure S14D (UNT-HDAC8 blotting)

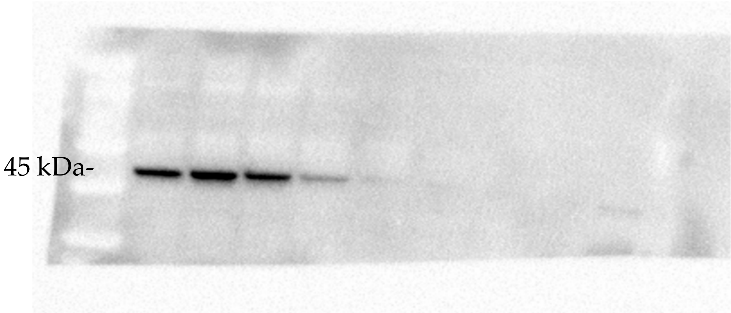

Figure S14D (GSK2879552-HDAC8 blotting)

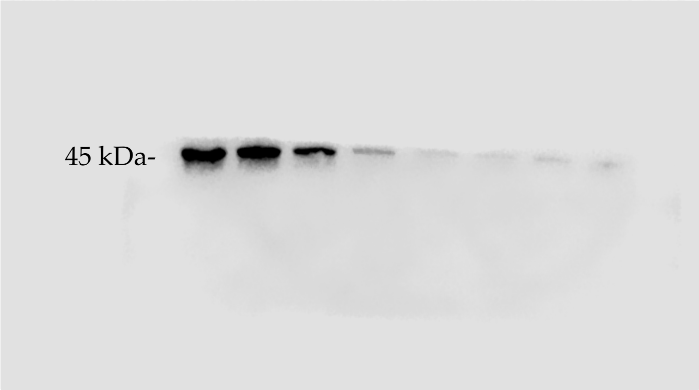

Figure S14D (Ricolinostat-HDAC8 blotting)

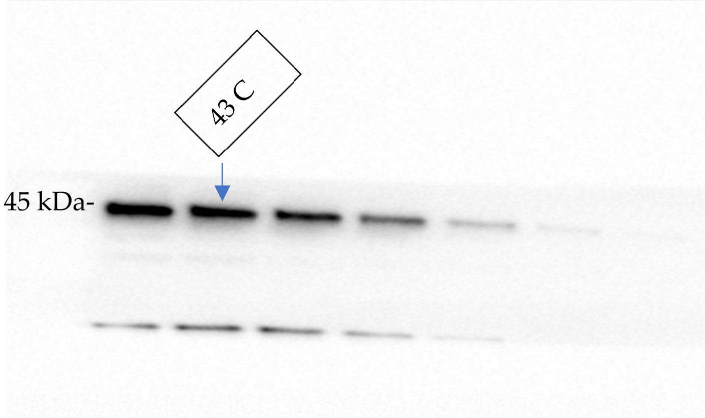

Figure S14D (iNC-HDAC8 blotting)

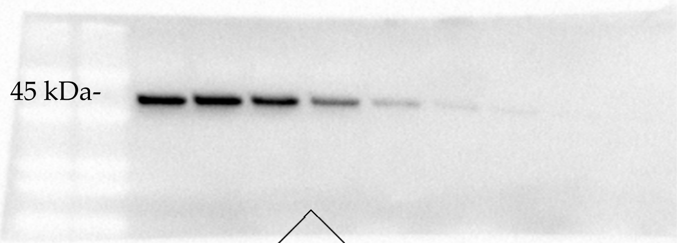

Figure S14D (iHDAC6-HDAC8 blotting)

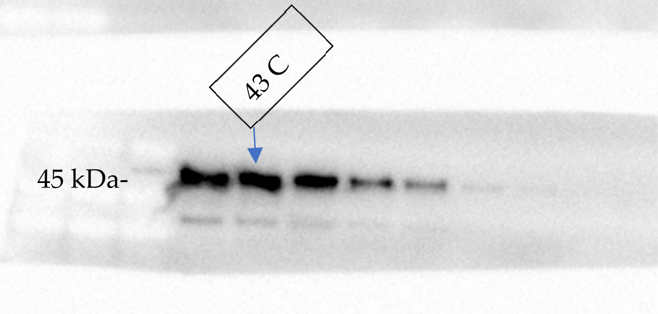

Figure S14D (iDual-HDAC8 blotting)

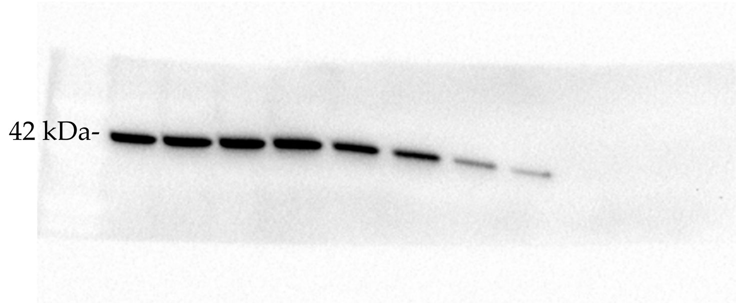

Figure S14D (Beta actin blotting)

Figure S31. Uncropped Western Blot images from Figure S14.

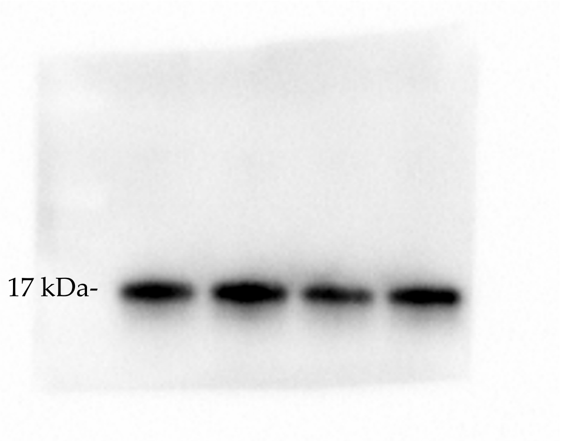

Figure S18A (H3K4me)

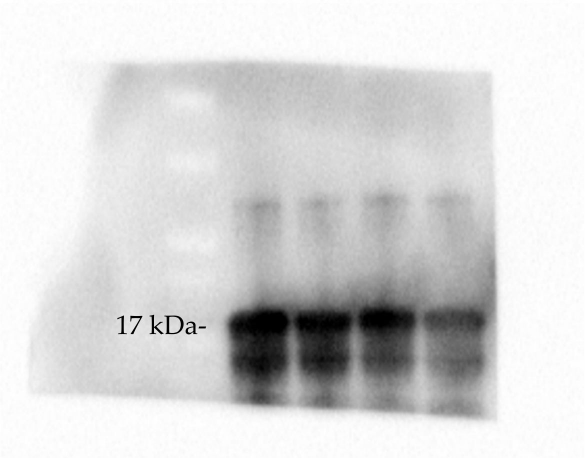

Figure S18A (H3 Total)

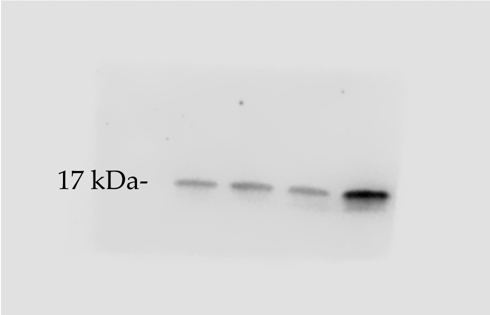

Figure S18B (H3K18ac)

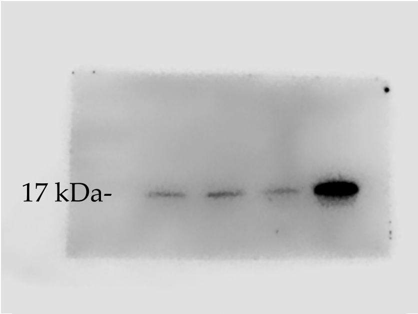

Figure S18B (H3K27ac)

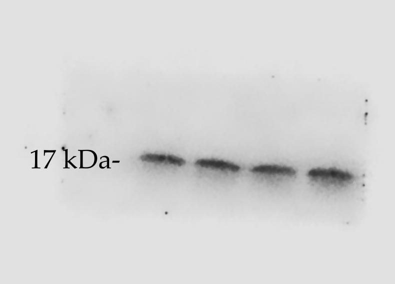

Figure S18B (H3 Total)

Figure S32. Uncropped Western Blot images from Figure S18.

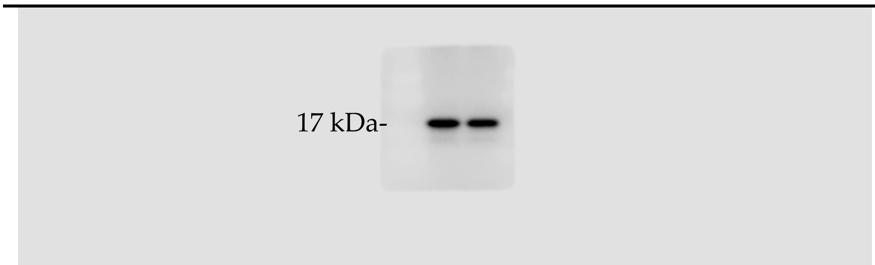	Figure S19 (H3K18ac)
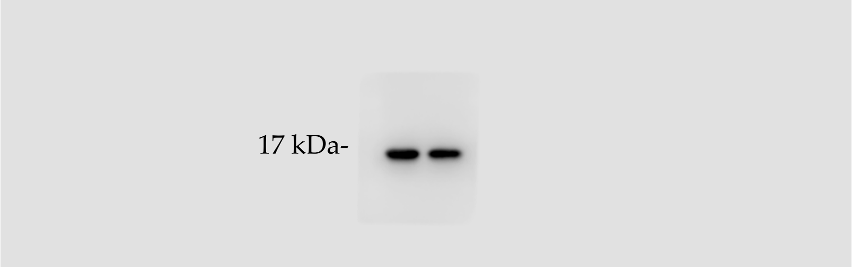	Figure S19 (H3K27ac)
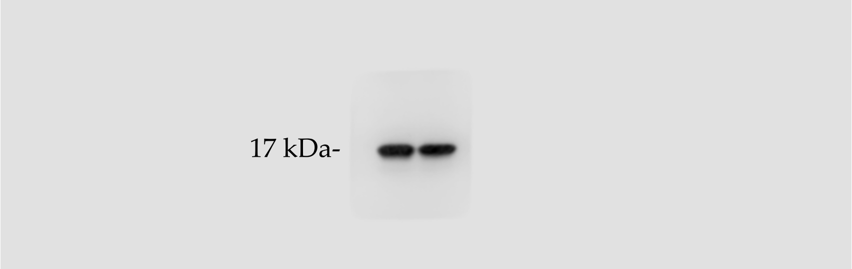	Figure S19 (H3 Total)

Figure S33. Uncropped Western Blot images from Figure S19.
